# A Cobalt@Cucurbit[5]uril Complex as a Highly Efficient Supramolecular Catalyst for Electrochemical and Photoelectrochemical Water Splitting

**DOI:** 10.1002/anie.202011069

**Published:** 2020-11-24

**Authors:** Fusheng Li, Hao Yang, Qiming Zhuo, Dinghua Zhou, Xiujuan Wu, PeiLi Zhang, Zhaoyang Yao, Licheng Sun

**Affiliations:** ^1^ State Key Laboratory of Fine Chemicals Institute of Artificial Photosynthesis, DUT-KTH Joint Education and Research Centre on Molecular Devices Dalian University of Technology 116024 Dalian China; ^2^ Department of Chemistry School of Engineering Sciences in Chemistry, Biotechnology and Health KTH Royal Institute of Technology 10044 Stockholm Sweden; ^3^ Center of Artificial Photosynthesis for Solar Fuels School of Science Westlake University 310024 Hangzhou China

**Keywords:** electrocatalysis, host–guest complexes, PEC cells, supramolecular catalysts, water splitting

## Abstract

A host–guest complex self‐assembled through Co^2+^ and cucurbit[5]uril (Co@CB[5]) is used as a supramolecular catalyst on the surface of metal oxides including porous indium tin oxide (ITO) and porous BiVO_4_ for efficient electrochemical and photoelectrochemical water oxidation. When immobilized on ITO, Co@CB[5] exhibited a turnover frequency (TOF) of 9.9 s^−1^ at overpotential *η*=550 mV in a pH 9.2 borate buffer. Meanwhile, when Co@CB[5] complex was immobilized onto the surface of BiVO_4_ semiconductor, the assembled Co@CB[5]/BiVO_4_ photoanode exhibited a low onset potential of 0.15 V (vs. RHE) and a high photocurrent of 4.8 mA cm^−2^ at 1.23 V (vs. RHE) under 100 mW cm^−2^ (AM 1.5) light illumination. Kinetic studies confirmed that Co@CB[5] acts as a supramolecular water oxidation catalyst, and can effectively accelerate interfacial charge transfer between BiVO_4_ and electrolyte. Surface charge recombination of BiVO_4_ can be also significantly suppressed by Co@CB[5].

## Graphical abstract

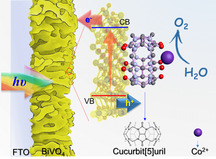

## Introduction

Photoelectrochemical (PEC) water splitting is a promising strategy for converting solar energy into renewable fuels, such as hydrogen,^[1]^ however, efficient catalytic water oxidation to provide protons for hydrogen production is considered to be a key challenge and one of the major obstacles for overall water splitting.^[2]^ The sluggish multi‐electron and multi‐proton processes involved in the water oxidation reaction made many semiconductors having a low catalytic activity towards the oxygen evolution reaction (OER). Thus, it is well accepted that semiconductors should be integrated with water oxidation catalysts to achieve efficient solar water splitting.^[3]^

Recent studies have reported considerable improvements in the OER catalyzed by non‐precious metal‐based catalysts such as cobalt, iron and nickel in the form of oxide, hydroxide and alloy with activity and stability benchmarks surpassing those of RuO_2_ and IrO_2_ under alkaline conditions.^[4]^ Bismuth vanadate (BiVO_4_) showed great prospect as a photoanode material to be coupled with catalysts for OER, due to its appropriate band structure.^[5]^ BiVO_4_ has been coupled with various OER material catalysts, such as Co_3_O_4_,^[6]^ FeOOH/NiOOH,^[7]^ FeOOH,^[8]^ CoPi,^[9]^ NiBi^[10]^ and NiB alloy.^[11]^ However, these heterogeneous catalysts for OER on the electrode surface are generally prepared through hydrothermal, electrodeposition or photo‐electrodeposition methods. In such processes, the thickness of the catalyst film has to be carefully controlled because it considerably influences the light absorption and the charge transport, therefore, ultra‐thin layers of catalysts are beneficial for efficient catalyst‐semiconductor hybrid photoanodes.[^2^, ^8^] Apart from heterogeneous OER catalysts coupled with semiconductors, molecular catalysts have also attracted great attention owing to their high activity, structural diversity, and facility in mechanistic studies.[^12^, ^13^] However, few efforts have been made to apply host–guest supramolecular complexes as molecular catalysts for semiconductor‐based photoanodes.

Cucurbituril (CB[*n*]) is a kind of macrocyclic host compounds, which can readily coordinate with metal cations as carbonyl groups directed towards the cavity of its cyclic structure. Strong complexes can be formed with cations by ion‐dipole interactions.[^14^, ^15^] Furthermore, CB[*n*] can be anchored or physiosorbed onto the surface of metal oxides (MO_x_),[^16^, ^17^] which indicates that the host–guest complexes based a CB[*n*] and a metal cation could be immobilized onto the surface of a metal oxide‐based semiconductor through the remaining carbonyl groups. In the CB[*n*] family, CB[5] and CB[7] are moderately soluble in water (with concentration of 20–30 mM),^[18]^ which is favorable for the construction of water splitting devices. In the present work, we selected CB[5] and Co^2+^ cation for the supramolecular assembly, and immobilized Co@CB[5] on the surface of metal oxides to study its activity for electrochemical and PEC water splitting. For the first time, we could demonstrate a host–guest complex Co@CB[5], which is applied to a porous ITO or BiVO_4_ surface, achieving the electrochemical and PEC water oxidation performance comparable with that of state‐of‐the‐art heterogeneous water oxidation catalysts, but with a much simpler preparation procedure. These new findings show the huge potential of host–guest supramolecular complexes as catalysts for efficient OER.

## Results and Discussion

### Electrode Preparation and Characterization

The Co@CB[5] supramolecular complex was assembled on the porous indium tin oxide (ITO) and porous BiVO_4_ substrates to evaluate the electrochemical‐driven and PEC oxygen evolution activity of the proposed host–guest assembled devices, respectively. The porous ITO substrate was prepared by a doctor‐blade method (see the Supporting Information for details). The BiVO_4_ thin film was prepared by modifying a method reported by Choi and co‐workers.^[19]^ This porous BiVO_4_ film has the advantages of improving electron‐hole separation and suppressing bulk carrier recombination,^[12]^ while also providing a large specific surface area for loading the Co@CB[5] onto the electrode surface. The porous ITO substrate was composed of octahedron shape particles as indicated by scanning electron microscope (SEM) images (Figure S1a). A cross‐sectional view of the film showed that the porous ITO layer had an average thickness of approximately 1.3 μm (Figure S1b). The porous BiVO_4_ film was composed of worm‐like particles as indicated by SEM images (Figure S2a). A cross‐sectional view of the film showed that the BiVO_4_ layer had an average thickness of approximate 1 μm (Figure S2b). Energy dispersive spectroscopy (EDS) mapping analysis was performed on a selected cross‐sectional area to determine the elemental distribution (Figure S2c), which demonstrated a homogeneous distribution of Bi, V, and O elements in the film of the porous BiVO_4_.

**Scheme 1:**
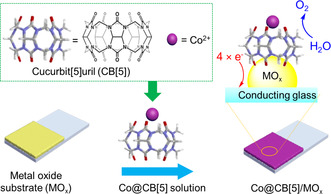
The electrode preparation process by immobilizing Co@CB[5] on the surface of metal oxide substrates.

The preparation of Co@CB[5]/ITO and Co@CB[5]/BiVO_4_ electrodes was briefly illustrated in Scheme 1. First, a solution of Co@CB[5] host–guest complex (1.0 mM) was prepared by mixing CB[5] and Co(NO_3_)_2_ in a molar ratio of 1:1. Mass‐to‐charge ratio (m/e) of 454.0879 corresponding to Co@CB[5]+H_3_O (m/e 454.0979) could be found by electrospray mass spectrometry (Figure S3). Then, the porous ITO and BiVO_4_ substrates were immersed in the solution of Co@CB[5] for 30 min, after washing with deionized water, resulting in the generation of Co@CB[5]/ITO and Co@CB[5]/BiVO_4_ electrodes, respectively. The attenuated total reflection infrared (ATR‐IR) spectra of Co@CB[5]/ITO, CB[5]/ITO electrode and bare CB[5] powder were acquired using an infrared spectrometer with a resolution of 0.09 cm^−1^ (Figure S4). Red‐shifts of ca. 9 and ca. 3 cm^−1^ were observed for the carbonyl portal of the CB[5] component on Co@CB[5]/ITO and CB[5]/ITO electrode compared to that of CB[5] powder (1738 cm^−1^), respectively. A similar red‐shift on the carbonyl portal of the CB[5] component on Co@CB[5]/BiVO_4_ and CB[5]/BiVO_4_ could also be observed (Figure S5). These ATR‐IR spectra suggested strong interaction between CB[5], Co cations and porous MO_x_ substrates,^[20]^ and indicated that Co@CB[5] was immobilized on the surface of ITO and BiVO_4_.

A time‐of‐flight secondary ion mass spectrometry (TOF‐SIMS) with Bi_3_^+^ as the primary ion source was further employed to detect the presence of Co@CB[5] on the surface of ITO and BiVO_4_. As shown in the SIMS images of Co@CB[5]/ITO and Co@CB[5]/BiVO_4_ (Figures S6 and S7), some related secondary ions assigned to cobalt combined with the fragments of CB[5] could be observed uniformly distributing on the surface of electrodes, indicating that Co@CB[5] was successfully immobilized on the surface of ITO and BiVO_4_.

The morphological features of Co@CB[5] functionalized electrodes were then investigated by SEM. As shown in Figures S8 and S9, no obvious morphological changes were observed after the immobilization of CB[5] and Co@CB[5] on the porous substrates, because the assembly process is a molecular level surface modification.

**Figure 1:**
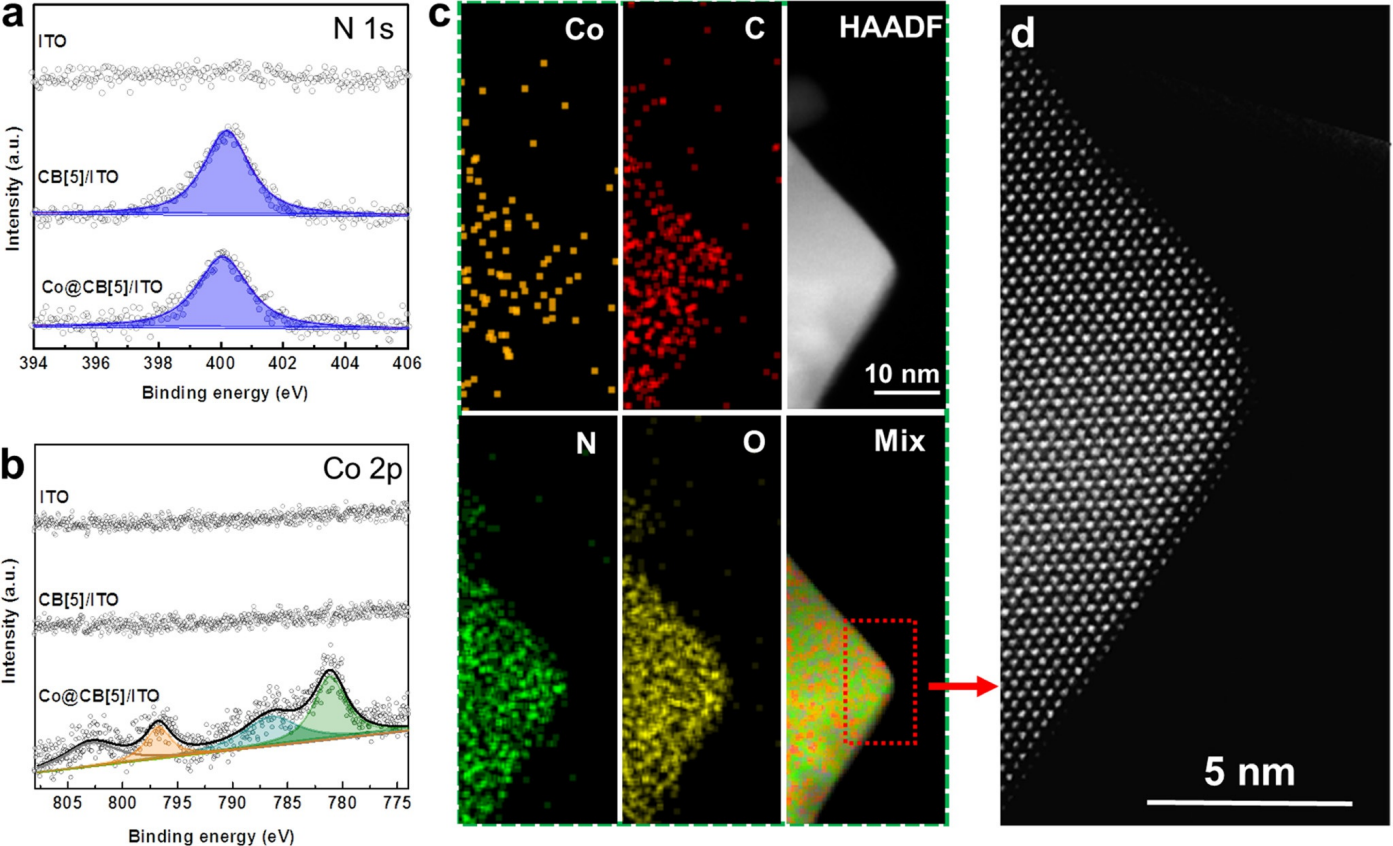
XPS spectra of ITO based electrodes in selected energy areas: a) N 1s, b) Co 2p. c) Energy‐dispersive X‐ray spectroscopy (EDS) maps of one Co@CB[5]/ITO particle. d) Atomic‐resolution HAADF image of one Co@CB[5]/ITO particle in selected small area.

**Figure 2:**
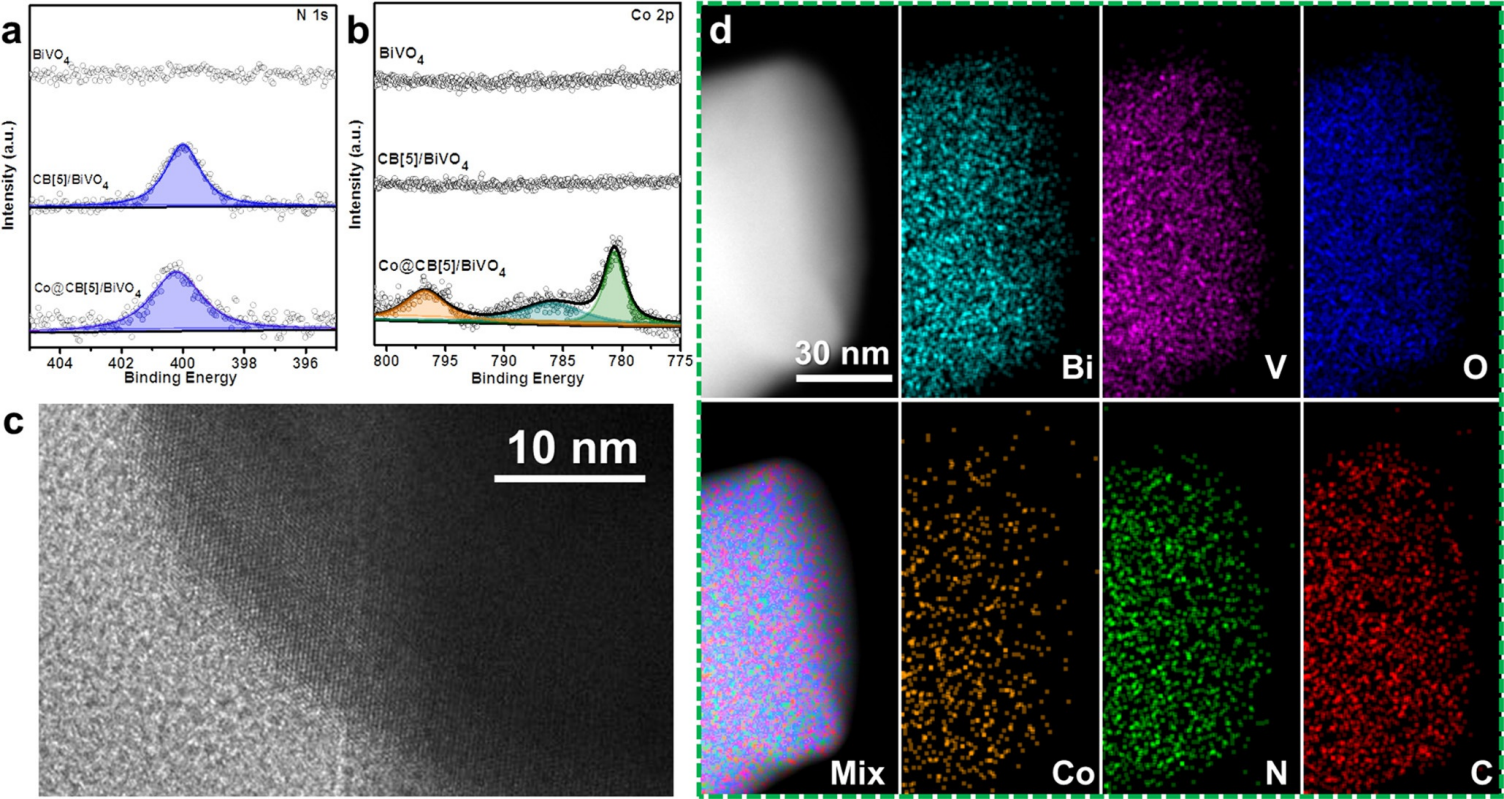
High‐resolution XPS spectra of BiVO_4_ based electrodes: a) N 1s and b) Co 2p. c) HRTEM image of the Co@CB[5]/BiVO_4_ sample. d) HAADF‐STEM images of Co@CB[5]/BiVO_4_ electrode and corresponding elemental mappings.

X‐ray photoelectron spectroscopy (XPS) measurements were performed to analyze the molecular level surface composition and electronic states of the Co@CB[5] complex functionalized MO_x_ substrates. The XPS survey spectra of Co@CB[5]/ITO, Co@CB[5]/BiVO_4_ and reference electrodes are shown in Figures S10 and S11. In XPS high‐resolution spectra of CB[5]/ITO and Co@CB[5]/BiVO_4_ (Figures 1 a,b, Figures 2 a,b), when CB[5] was immobilized on the surface of MO_x_, the N 1s signal corresponding to the C‐N of CB[5] at a binding energy of 400.15 eV can be observed, which indicated that CB[5] can be successfully immobilized on the surface of porous MO_x_ substrates by the simple soaking method. When Co@CB[5] was immobilized on ITO or BiVO_4_, clear signal of Co 2p were observed at the binding energy around 781 eV, which did not appear for the bare substrates and neither for the CB[5]/MO_x_ electrodes. Meanwhile, small offsets from the main signal of N 1s could be observed. The signal of Co 2p for Co@CB[5]/ITO was fitted by four peaks, namely Co 2p_3/2_ at 781.0 eV, Co 2p_3/2_ satellite at 786.3 eV, Co 2p_1/2_ at 796.7 eV, and a Co 2p_1/2_ satellite at 802.6 eV. The signal of Co 2p for Co@CB[5]/BiVO_4_ was fitted into three peaks, Co 2p_3/2_ at 780.7 eV, a Co 2p_3/2_ satellite at 785.9 eV, and Co 2p_1/2_ at 796.6 eV, (the Co 2p_1/2_ satellite signal is not assigned here owing to its overlap with the BiVO_4_ signal). Please note that the binding energies of Co 2p for Co@CB[5] complex were different from those of Co‐based oxides or hydroxides,[^21^, ^22^] indicating a molecular nature of the Co@CB[5] on MO_x_ surfaces.

To further demonstrate that the Co@CB[5] is a molecular catalyst on MO_x_, the surface and morphology properties of the Co@CB[5]/ITO and Co@CB[5]/BiVO_4_ samples were studied at atomic level by spherical aberration corrected transmission electron microscope (ACTEM). As revealed by high‐angle annular dark‐field (HAADF) images in Figure S12, the Co@CB[5]/ITO nano‐particle exhibited octahedron shape with regular and clear edge. As shown in Figure 1 c, Energy‐dispersive X‐ray spectroscopy (EDS) mapping images of Co@CB[5]/ITO showed the presence of Co, N and C on the surface of ITO nano‐particle. Atomic‐scale HAADF images in the Figure 1 d revealed that there is no structural decomposition observed after the attachment of Co@CB[5] complex, meanwhile, no Co oxides or hydroxides could be observed on the terminations of ITO nano‐particle after the immobilization of Co@CB[5] complex. This is in consistence with the phenomena of molecular anchoring of Co@CB[5] complex on ITO surface. As the element atomic mass of tin and indium in bulk ITO nanoparticle is far larger than that of cobalt, therefore, it is hard to pinpoint the single cobalt molecular complex absorbed on the surface of ITO by comparing the difference in atomic resolution HAADF images. Figures S13 and S14 have shown atomic‐resolution TEM images and corresponding EDS mapping images of Co@CB[5]/ITO in different scales, which suggested that the cobalt components were uniformly distributed on the surface of ITO. In addition, according to the results of EDS analysis in selected area, the metal composition ratio of Co: In: Sn was estimated to be 1: 92: 7, this result was in accordance with the magnitude of surface mono‐molecular layer distribution for Co components.^[23]^

The interface morphology of Co@CB[5]/BiVO_4_ was characterized by high‐resolution transmission electron microscope (HRTEM), as shown in Figure 2 c, there were no obvious metal oxide films or nanoparticles covered on the surface of Co@CB[5]/BiVO_4_ nanoparticle. Unfortunately, when high energy electron beam was focused on BiVO_4_, the surface of BiVO_4_ was damaged, detailed structure information for the surface of Co@CB[5]/BiVO_4_ could not be obtained by atomic resolution AC‐STEM. HAADF‐STEM and corresponding EDS mapping images of Co@CB[5]/BiVO_4_ showed that elements of Co, N and C contents in the Co@CB[5] complex were uniformly distributed on the surface of the BiVO_4_ nanoparticles (Figures 2 d and S15). All above characterizations suggested that the Co@CB[5] on the surface of MO_x_ is a molecular level immobilization.

### Electrochemically Driven OER Catalysis by the Co@CB[5] Complex

**Figure 3:**
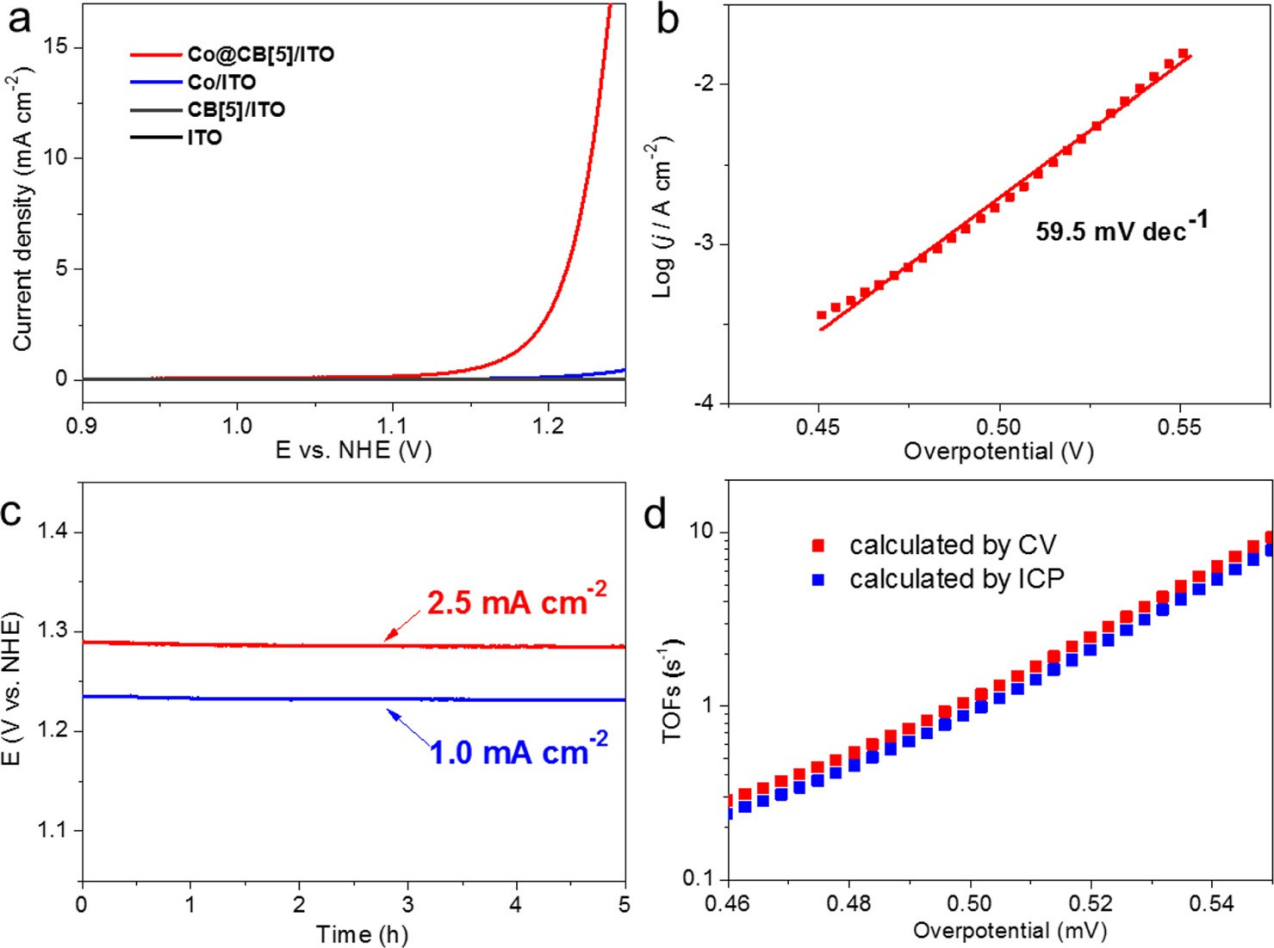
a) The LSV measurements of Co@CB[5]/ITO and comparison electrodes in 1.0 M borate buffer (pH 9.2) at a scan rate of 50 mV s^−1^ with *iR* compensation. b) Tafel plots of Co@CB[5]/ITO. c) Chronopotentiometric measurement of OER at current density of 1.0 and 2.5 mA cm^−2^ for 5 h without *iR* compensation. d) TOFs of Co@CB[5]/ITO based on the Co^2+^ loading (calculated according to electrochemical measurement and ICP‐OES) and current densities vs. various overpotentials.

The OER performance of Co@CB[5]/ITO was first evaluated by linear sweep voltammetry (LSV). LSV measurements were performed at 25 °C in a standard three‐electrode cell with 1.0 M borate buffer solution (pH 9.2) as the electrolyte. As shown in Figure 3 a, the ITO substrate and CB[5]/ITO electrode were silent for OER. In contrast, the anodic current density curve of the Co@CB[5]/ITO electrode rose sharply beyond a low onset potential of 1.05 V vs. normal hydrogen electrode (NHE), followed by a dramatic increase at more positive potentials. A current density of 1.0 mA cm^−2^ was achieved at *η*=485 mV. For comparison, Co/ITO electrode was also prepared by immersing ITO in 1.0 mM Co^2+^ solution for 30 mins (the same immersion time as for Co@CB[5]/ITO). The Co/ITO electrode exhibited a much lower anodic current density than that of the Co@CB[5]/ITO electrode. Linear fitting of the Co@CB[5]/ITO electrode gives a Tafel slope of 59.5 mV dec^−1^ (Figure 3 b). The steady‐state catalytic activities of Co@CB[5]/ITO were determined by chronopotentiometric measurements during electrolysis at constant current densities over 5 h (Figure 3 c). The Co@CB[5]/ITO electrode required only 1.23 V and 1.29 V vs. NHE to achieve the current density of 1.0 mA cm^−2^ and 2.5 mA cm^−2^, respectively. The overpotential requirement kept stable during the test, which indicated that the Co@CB[5]/ITO is stable for OER. The amount of electrochemically generated oxygen from the Co@CB[5]/ITO system was confirmed by gas chromatography (GC). When a total charge of 3.6 C passed through the electrode, 8.62 μmol of O_2_ was detected by GC, leading to a Faraday efficiency of 92.6 % (Figure S16).

The effective coverage of Co^2+^ on the surface of ITO was estimated as 4.54×10^−9^ mol cm^−2^ according to the linear relationship between the peak current of Co^2+/3+^ and the scan rate (Figure S17, Eq. S1 and Eq. S2), and the order of magnitude for such catalyst loading is consistent with that of molecules immobilized on the surface of porous metal oxide substrates.^[24]^ This total loading amount of Co^2+^ was further confirmed by inductively coupled plasma optical emission spectrometer (ICP‐OES). The total Co^2+^ loading amount is 4.98×10^−9^ mol cm^−2^, which was slightly larger than the redox active Co^2+^. The turn over frequencies (TOFs) of Co@CB[5]/ITO based on the Co^2+^ loading (redox active) current densities vs. various overpotentials were calculated based on LSV data (operated in a low scan rate of 10 mV s^−1^) and Eq. S3.^[25]^ The logarithm of the TOFs varied linearly on the applied overpotentials from 460 to 550 mV, as shown in Figure 3 d. A TOF of 0.3 s^−1^ was achieved at *η*=460 mV, which reached 9.9 s^−1^ at *η*=550 mV. Table S1 lists the performance of Co@CB[5]/ITO in the current work and previously reported anodes for electrocatalytic water oxidation, including molecular and inorganic material catalysts immobilized on conducting glass. This comparison shows that the Co@CB[5]/ITO electrode is one of the best OER anodes in terms of overpotential, current density, and TOF.

### Photo‐electrochemical Water Oxidation by Co@CB[5]/BiVO_4_

The PEC water oxidation performance of Co@CB[5]/BiVO_4_ photoanode was measured by linear sweep voltammetry (LSV) with a scan rate of 10 mV s^−1^. The absorption spectrum of BiVO_4_ did not change after Co@CB[5] was immobilized on the surface of BiVO_4_ (Figure S18), hence, the band gap of BiVO_4_ and Co@CB[5]/BiVO_4_ were the same.

**Figure 4:**
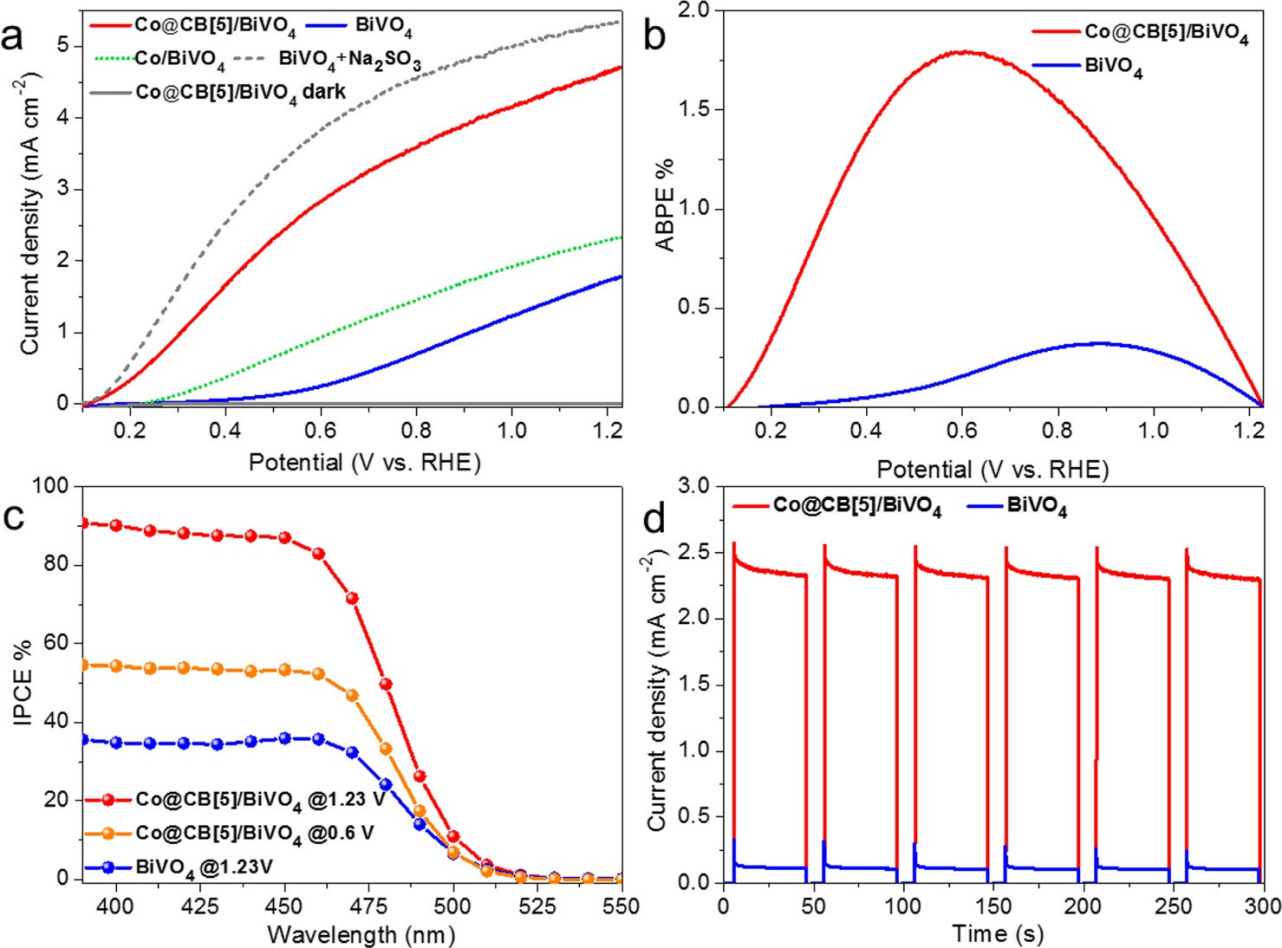
a) LSV curves of Co@CB[5]/BiVO_4_, Co/BiVO_4_, BiVO_4_, and BiVO_4_ with the hole scavenger Na_2_SO_3_ in 1 M borate buffer (scan rate, 10 mV s^−1^). b) ABPEs of Co@CB[5]/BiVO_4_ and BiVO_4_ calculated from LSV curves. c) IPCEs of Co@CB[5]/BiVO_4_ at 1.23 V and 0.6 V vs. RHE, and BiVO_4_ at 1.23 V vs. RHE. d) Light response of Co@CB[5]/BiVO_4_ and BiVO_4_ photoanodes under chopped irradiation at a constant bias of 0.6 V vs. RHE.

As shown in Figure 4 a, the photocurrent density of the as‐prepared BiVO_4_ at 1.23 V vs. RHE was approximate 1.8 mA cm^−2^. In the presence of sulfite as hole scavenger, the photocurrent density of BiVO_4_ increased to 5.4 mA cm^−2^. These results are comparable with previously reported values,^[26]^ which suggested that the quality of BiVO_4_ film in our work is reliable. When Co@CB[5] was immobilized on the surface of BiVO_4_, the photocurrent density of the Co@CB[5]/BiVO_4_ electrode was measured to be 4.8 mA cm^−2^ at 1.23 V vs. RHE without the hole scavenger sulfite, which is much greater than that of unmodified BiVO_4_, CB[5]/BiVO_4_ and Co/BiVO_4_ electrodes. The CB[5]/BiVO_4_ electrode exhibited an similar anodic photocurrent density curve than that of the pristine BiVO_4_ electrode (Figure S19), indicating CB[5] itself can not affect the water oxidation of BiVO_4_. After immersing BiVO_4_ in Co^2+^ solution for 30 minutes, the photocurrent density of Co/BiVO_4_ electrode at 1.23 V vs. RHE increased to 2.3 mA cm^−2^, which is much lower than that of the Co@CB[5]/BiVO_4_ electrode (4.8 mA cm^−2^). The applied bias photon to current efficiency (ABPE) was calculated from the corresponding LSV curve in Figure 4 a according to Eq. S4. The maximum ABPE of BiVO_4_ was 0.32 % at 0.88 V vs. RHE. However, the maximum ABPE of Co@CB[5]/BiVO_4_ was 1.79 % at 0.60 V vs. RHE (Figure 4 b), which is 5.5 times as high as that of unmodified BiVO_4_. The incident photon to current efficiencies (IPCEs) of BiVO_4_ and Co@CB[5]/BiVO_4_ were measured and calculated according to Eq. S5. As shown in Figure 4 c, the IPCE values of BiVO_4_ and Co@CB[5]/BiVO_4_ were approximate 35 % and 88 % at 420 nm (1.23 V vs. RHE), respectively. The values of the ABPE and IPCE demonstrated the high photon‐to‐O_2_ conversion efficiency of the host–guest complex assembled Co@CB[5]/BiVO_4_ photoanode. On the basis of these measurements, the photocurrent densities were 4.85 mA cm^−2^ at 1.23 V vs. RHE and 2.9 mA cm^−2^ at 0.6 V vs. RHE for Co@CB[5]/BiVO_4_, as estimated by integrating the IPCE curves over the AM 1.5G solar spectrum (Figure S20). These calculated values are in agreement with the measured data in Figure 4 a (4.8 mA cm^−2^ at 1.23 V and 2.8 mA cm^−2^ at 0.6 V).

Under chopped‐light illumination, the bare BiVO_4_ only generated a low photocurrent density less than 0.25 mA cm^−2^ at a constant applied potential of 0.6 V vs. RHE; however, the Co@CB[5]/BiVO_4_ photoanode generated a photocurrent density of ca. 2.4 mA cm^−2^, underlining the indispensable role of Co@CB[5] in water splitting (Figure 4 d). During long‐term photolysis at 0.6 V vs. RHE under continued visible‐light irradiation, the magnitude of the photocurrent generated by Co@CB[5]/BiVO_4_ slowly decreased from 2.5 to 2.3 mA cm^−2^ over 30 min (Figure S21). The photogenerated O_2_ in the headspace was quantified by GC. Assuming a 4 e^−^ process for O_2_ evolution, the number of electrons passing through the electrode agreed well with the amount of O_2_ detected, representing a Faradaic efficiency of 90.3 % for Co@CB[5]/BiVO_4_ photoanode (Figure S22).

The photocurrent density at 1.23 V vs. RHE and the maximum ABPE obtained from Co@CB[5]/BiVO_4_ are compared to those previously reported systems and the results are shown in Table S2. The performance of Co@CB[5]/BiVO_4_ exceeded those of many catalyst‐modified undoped‐BiVO_4_ photoanodes in both parameters. The host–guest complex assembled Co@CB[5]/BiVO_4_ photoanode can avoid complicated organic synthesis, and the high activity of Co@CB[5]/BiVO_4_ holds potential for supramolecular catalysts capable of replacing state‐of‐the‐art metal oxides and molecular catalysts.

### Post‐characterization of the Electrode after the OER Test

Post‐characterization of the electrode after the OER test is necessary for further proof of reaction mechanism and the stability of the as‐fabricated hybrid electrodes. As shown in ATR‐IR spectra (Figure S23), Co@CB[5] maintained on the surface of electrodes after the OER test. As shown in Figures S24 and S25, the SEM images revealed that the Co@CB[5]/ITO and Co@CB[5]/BiVO_4_ after OER test maintained a porous surface without significant changes. The composition and electronic states of the Co@CB[5]/ITO and Co@CB[5]/BiVO_4_ samples after OER test were analyzed by XPS technique (Figures S26–S29). The binding energies of both N 1s and Co 2p for Co@CB[5]/ITO and Co@CB[5]/BiVO_4_ were consistent with that of before OER test, indicating that Co@CB[5] was stable for OER. The EDS elemental mapping images with different magnitudes in Figures S30a, S31 and S32 confirmed the uniform distribution of Co, N and C in Co@CB[5]/ITO following the OER tests. Figure S30b showed the atomic‐resolution TEM images of tested Co@CB[5]/ITO particle. By further observing the edge of sample under greater magnification, a clear and well‐ordered surface could be observed of tested Co@CB[5]/ITO, indicating that there was no other amorphous layer or heterojunction film formed to covered ITO nano‐particle surface during the OER process (Figure S30c,d). In addition, according to the results of EDS analysis in selected area, the metal composition ratio of Co: In: Sn for tested Co@CB[5]/ITO particle was calculated to be 1: 91: 8, which was almost the same as the ratio of pristine Co@CB[5]/ITO. The EDS elemental mapping images with different magnitudes in Figure S33 and S34 confirmed the uniform distribution of Co, N and C in Co@CB[5]/BiVO_4_ after the OER tests. Figure S35 showed the HRTEM images of the tested Co@CB[5]/BiVO_4_ particles, there are no nanoparticles generated on the surface of Co@CB[5]/BiVO_4_ during the OER process, indicating that Co@CB[5] was stable on the surface of BiVO_4_ during the OER. By integrating the information obtained from the above characterizations, it can be convincingly concluded that Co@CB[5] maintains its supramolecular nature and is stable during the OER.

### Kinetic Studies of Co@CB[5] for Water Oxidation

To investigate the transfer of carriers in the PEC system constructed by this novel Co@CB[5] supramolecular catalyst, kinetic studies were carried out. With sodium sulfite (Na_2_SO_3_) as a hole scavenger, extremely fast oxidation kinetics and negligible surface recombination were expected, as shown in Figure S36, the charge transfer efficiency (*η_trans_*) at the electrode‐electrolyte interface was calculated according to Eq. S6 and the data from Figure 4 a. Using this method, a superior *η_trans_* of Co@CB[5]/BiVO_4_ was achieved (88 %), compared with that of bare BiVO_4_ (33 %) at 1.23 V vs. RHE. This result suggests that the Co@CB[5]/BiVO_4_ electrode has more rapid water oxidation kinetics than the bare BiVO_4_.^[27]^

**Figure 5:**
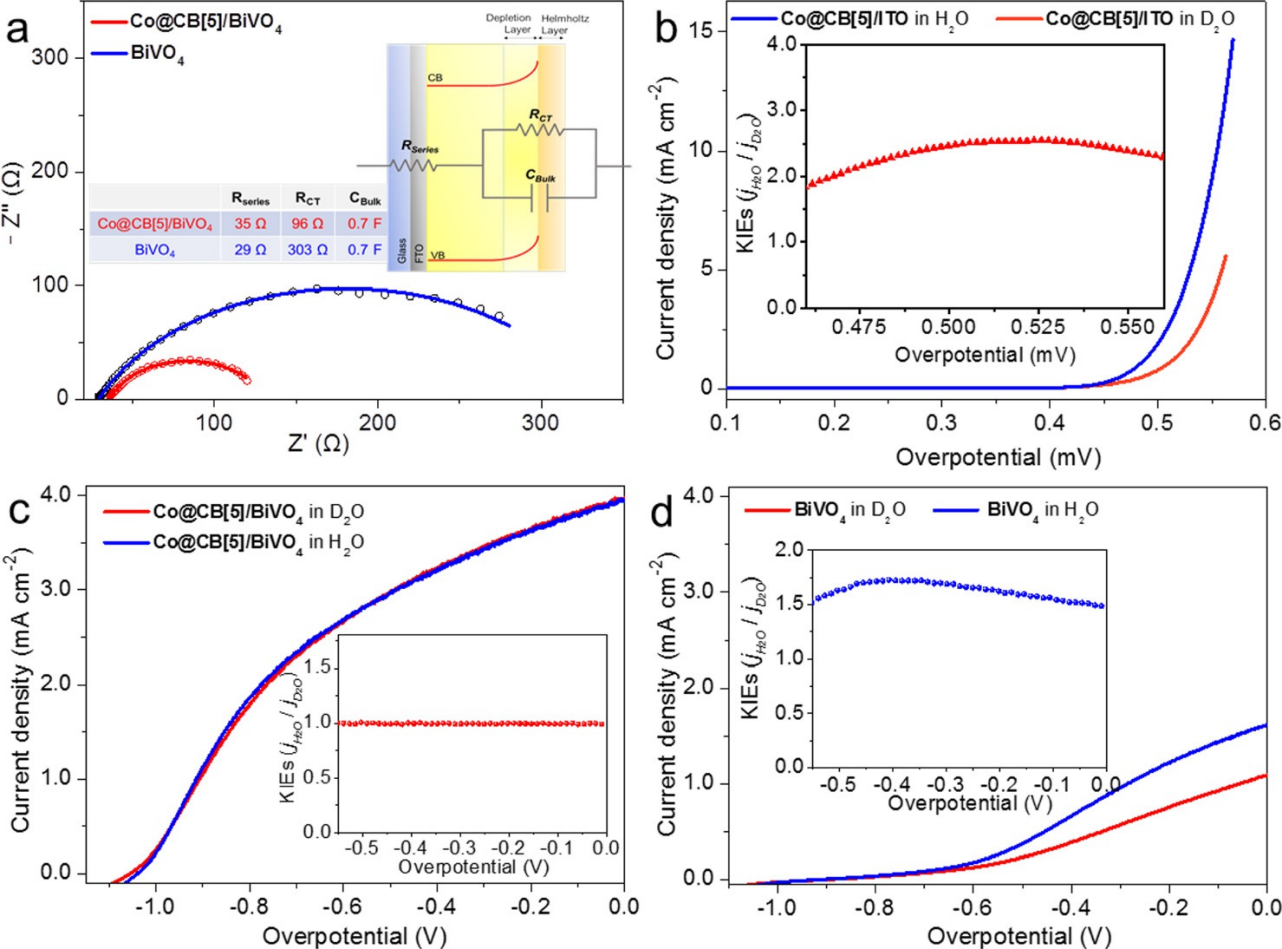
a) EIS of BiVO_4_ and Co@CB[5]/BiVO_4_ photoanodes measured under 0.6 V vs. RHE and AM 1.5G (100 mW cm^−2^) in 1.0 M borate buffer (pH 9.2). b) LSV curves of Co@CB[5]/ITO in anhydrous sodium borate H_2_O and D_2_O solutions, the inset exhibits the KIEs against potential. c),d) LSV curves of Co@CB[5]/BiVO_4_ (c) and BiVO_4_ (d) in anhydrous sodium borate H_2_O and D_2_O solutions under light irradiation, the inset exhibits the corresponding KIEs against potential.

Electrochemical impedance spectroscopy (EIS) was performed to examine the charge transport phenomena in the bulk and at the surface of the electrodes. As shown in Figure 5 a, semicircles for Co@CB[5]/BiVO_4_ and BiVO_4_ electrodes fitted well with Randles equivalent circuit model. Charge transport at the photoanode‐electrolyte interface was estimated with the values for the charge‐transfer resistance (R_ct_) obtained from the diameter of the semicircles. A smaller semicircle indicates better charge transfer of the corresponding photoanode at the interface. Notably, the Co@CB[5]/BiVO_4_ electrode had a markedly smaller R_ct_ (96 Ω) value than that of bare BiVO_4_ (303 Ω) under illumination at 0.6 V vs. RHE, illustrating a better ability to transfer holes at the electrode‐electrolyte interface of Co@CB[5]/BiVO_4_, which was consistent with its high charge transfer efficiency (*η_trans_*).

The charge transfer efficiency depends on the rate of water oxidization catalytic processes on the surface of BiVO_4_ and the rate of surface charge recombination. The deuterium kinetic isotope effects (KIEs) can reflect the proton transfer kinetic information on water oxidation reactions and help us to interpret the rate determine step (RDS) of the catalytic process.^[28]^ The KIEs(H/D) can be defined as Eq. S7, the current densities in water and deuterated water electrolytes were contrasted at the same overpotential according to Eq. S8–Eq. S10 for KIEs(H/D) measurements in this work.^[29]^

As shown in Figure 5 b, the value of KIEs of Co@CB[5]/ITO electrode was larger than 2, indicating a primary KIEs for which O−H bond cleavage is involved in the RDS of electro‐driven water oxidation for Co@CB[5], corresponding to the water nucleophilic attack mechanisms.^[28]^ When the Co@CB[5] was anchored on the surface of BiVO_4_, the value of KIEs for Co@CB[5]/BiVO_4_ photoanode was around 1.0 with the applied bias increasing, indicating the O−H bond cleavage is not involved in the RDS for light driven water oxidation by Co@CB[5]/BiVO_4_ photoanode (Figure 5 c). In contrast, the value of KIEs for bare BiVO_4_ photoanode was around 1.5 (Figure 5 d), suggesting that the proton transfer process was involved in the RDS of the light‐driven water oxidation process for bare BiVO_4_. Thereby, the immobilization of Co@CB[5] on the surface of BiVO_4_ could greatly accelerate the proton transfer processes (water oxidation) and shift the RDS of BiVO_4_ photoanode to a non proton transfer involved process. Thence, Co@CB[5] indeed played an important role in the light‐driven catalytic water oxidation processes in this Co@CB[5]/BiVO_4_ supramolecular assembly photoanode.

It has been known that a co‐catalyst can serve as passivation layer to improve charge‐separation and charge transfer processes across semiconductor‐liquid interfaces.^[30]^ For example, the modification of CoPi on the surface of semiconductors can improve the performances of photoanodes, which primarily caused by reducing the surface charge recombination (passivation layer), rather than improving the catalytic ability (charge transfer kinetics).[^31^, ^32^] Similar phenomena have also been observed in other systems, such as a thin layer of CoFeO_x_ modified hematite and BiFeO_3_ passivated BiVO_4_ photoanodes.[^33^, ^34^] Converse systems have also been reported, for instance, thin layers of FeOOH can enhance the performance of BiVO_4_ for water oxidation, for which the performance was improved primarily owing to the improvement of charge transfer, rather than suppressing the surface charge recombination.^11^ Molecular‐based catalysts can also improve the light‐driven water oxidation ability of n‐type semiconductors, such as Co‐salophen complexes and Ni_4_O_4_ cubane have been reported as the co‐catalyst for BiVO_4_,[^35^, ^36^] in these cases, molecular catalysts can accelerate the water oxidation reaction as OER catalysts, meanwhile, reduce the surface charge recombination.

**Figure 6:**
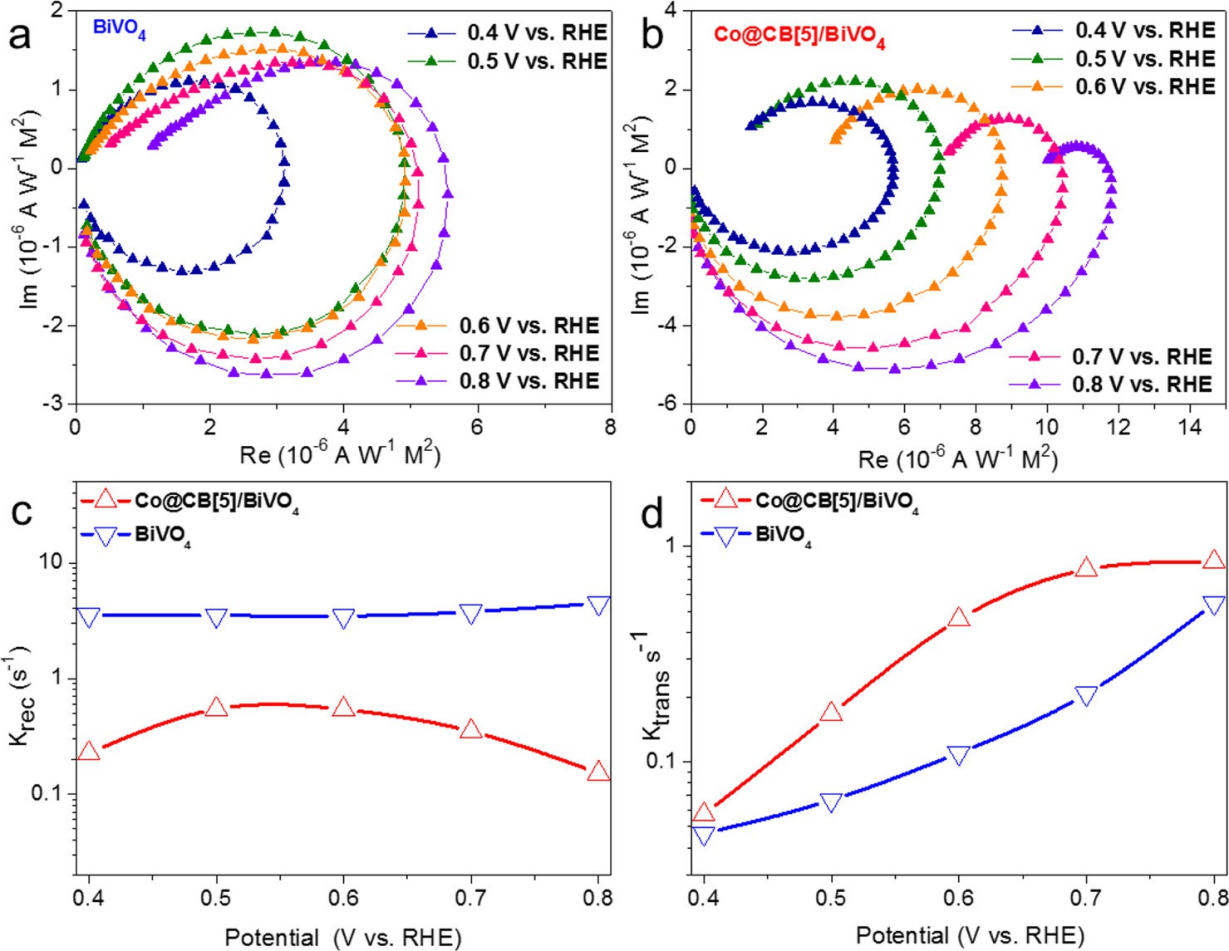
a) IMPS spectra of BiVO_4_ photoanode at various potentials. b) IMPS spectra of Co@CB[5]/BiVO_4_ photoanode at various potentials. c) Rate constant for charge recombination, *k*_rec_ extracted from IMPS spectra. d) Rate constant for charge transfer, *k*_trans_ extracted from IMPS spectra.

Our results confirmed that Co@CB[5] can effectively promote BiVO_4_ for light‐driven water oxidation by serving as a good OER catalyst, however, it is necessary to further confirm whether Co@CB[5] on the semiconductor inhibited surface charge recombination by acting as a surface passivation layer. The charge transfer and surface recombination kinetics were quantified by intensity modulated photocurrent spectroscopy (IMPS) to understand the real role of Co@CB[5] on the BiVO_4_. Figure 6 a and 6 b show typical IMPS responses of bare BiVO_4_ and Co@CB[5]/BiVO_4_ photoanodes in the complex plane. The IMPS spectrum consists of two semicircles in the 4th and 1st quadrants, which correspond to the resistance‐capacitance attenuation and the competition between charge transfer and recombination, respectively.^[34]^ Accordingly, the frequency of the maximum imaginary corresponds to the sum of the charge transfer (*k*_trans_) and charge recombination (*k*_rec_) rate constants (*k*_trans_+*k*_rec_=2π*f*_max_). The low frequency intercept in the normalized form, at which the imaginary part is equal to 0, corresponds to a charge transfer efficiency of *k*_trans_/(*k*_trans_+*k*_rec_).^[37]^ The key parameters *k*_rec_ and *k*_trans_ are therefore readily accessible.

Moreover, the upper semicircle of the BiVO_4_ photoanode did not change notably as the applied potential was increased, the value of *k*_rec_ remained nearly constant with increasing potential for bare BiVO_4_ (Figure 6 c), indicating that a high charge recombination exists for the BiVO_4_ photoanode over a wide potential range. By contrast, the upper semicircle of the Co@CB[5]/BiVO_4_ photoanode became smaller as the applied potential increased. Notably, *k*_rec_ was suppressed by a factor of 6.5–30 over the entire potential range after decoration of Co@CB[5]. For instance, at 0.6 V vs. RHE (the max point of ABPEs), the value of *k*_rec_ was 3.5 s^−1^ for bare BiVO_4,_ which is 6.5 times as large as that of Co@CB[5]/BiVO_4_ (0.54 s^−1^). At higher potentials, this factor increased to 30 at 0.8 V vs. RHE. These results suggest that the charge recombination was markedly suppressed by the Co@CB[5]. Figure 6 d shows that the *k*_trans_ of Co@CB[5]/BiVO_4_ surpassed that of BiVO_4_ at all potentials. Particularly, at a potential of 0.6 V vs. RHE (the max point of ABPEs), the *k*_trans_ of Co@CB[5]/BiVO_4_ was more than 4 times as great as that of BiVO_4_. The charge transfer efficiency, defined as *k*_trans_/(*k*_trans_+*k*_rec_), showed the same trend as the value determined by *J*_water_/*J*_sulfite_, indicating the reliability of our measurements (Figure S37). The ratio of *k*_rec_ */k*_trans_ was positively proportional to the semicircle in 1st quadrant and a small value indicated faster charge transfer than charge recombination.^[34]^ These results demonstrate that Co@CB[5] behaves as a molecular catalyst, which is in contrast to the mere passivation action of heterogeneous catalysts as reported,[^31^, ^32^, ^33^, ^34^] Co@CB[5] not only accelerates the water oxidation reaction as a good OER catalyst for BiVO_4_, but also reduces the surface charge recombination.

**Scheme 2:**
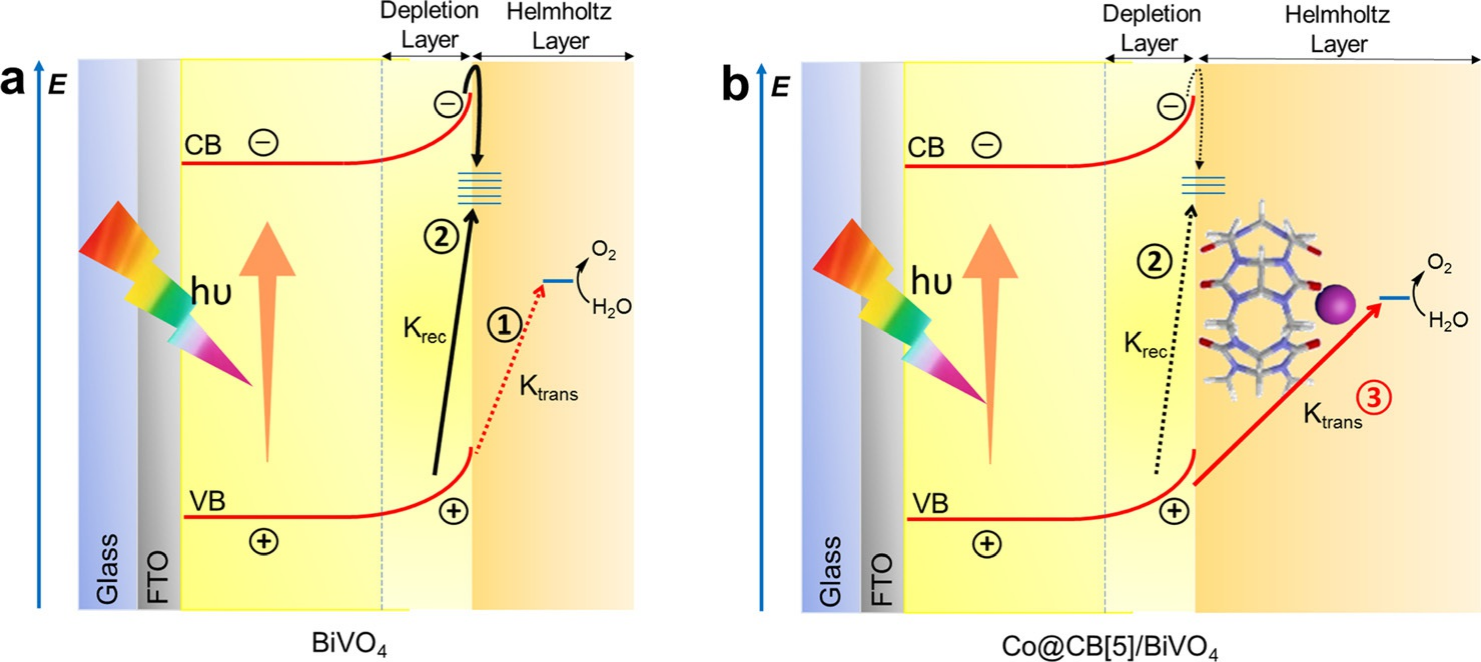
Simplified elementary processes in a) bare BiVO_4_ and b) Co@CB[5]/BiVO_4_ photoanode. The movement of holes that were generated from photo‐induced charge separation have three pathways at the semiconductor‐electrolyte interface (solid lines: majority events; dotted lines: minority events): 1) direct water oxidation with low *k*_trans_, 2) surface charge recombination, and 3) charge transfer via Co@CB[5] with high *k*_trans_.

Based on the OER kinetics analyzed above, the function of Co@CB[5] on BiVO_4_ was summarized and shown in Scheme 2. For the bare BiVO_4_, the photo generated holes can be directly consumed for water oxidation (pathway 1), or recombined with electrons that trapped by surface state (pathway 2). Due to the low intrinsic OER catalytic activity of BiVO_4_, the injection of holes to electrolyte was limited by the proton transfer involved water oxidation reaction, leading to a primary KIEs for bare BiVO_4_. When Co@CB[5] was immobilized on the surface of BiVO_4_, owing to the high OER catalytic activity of Co@CB[5], the photo‐generated holes could be effectively transferred and consumed for water oxidation (pathway 3); meanwhile, due to the faster consumption of holes, the photo‐generated electrons will have less chance to recombine with photo‐generated holes at the surface of BiVO_4_, leading to the higher charge transfer efficiency of Co@CB[5]/BiVO_4_ in comparison to that of the bare BiVO_4_.

## Conclusion

A host–guest supramolecular complex Co@CB[5] as a molecular water oxidation catalyst was successfully immobilized on porous ITO and BiVO_4_ substrates. This supramolecular catalyst Co@CB[5] showed a high activity for electrochemical water oxidation when immobilized on a porous ITO substrate, the fabricated Co@CB[5]/ITO electrode exhibited a TOF of 9.9 s^−1^ at an overpotential of 550 mV in a pH 9.2 borate buffer with good stability. When Co@CB[5] was immobilized on the surface of the porous BiVO_4_ n‐type semiconductor, the integrated photoanode Co@CB[5]/BiVO_4_ showed an excellent PEC performance with a high photocurrent density of 4.8 mA cm^−2^ at 1.23 V (vs. RHE) under 100 mW cm^−2^ (AM 1.5) light illumination. KIEs and IMPS results confirmed that Co@CB[5] serves as a supramolecular water oxidation catalyst which can effectively accelerate surface charge transfer. Furthermore, the surface charge recombination of BiVO_4_ can also be suppressed by this host–guest supramolecular complex. The host–guest complex Co@CB[5] shows a remarkable performance for electrochemical and PEC water oxidation, which can open new opportunities for the development of supramolecular complexes as catalysts for not only efficient water oxidation, but may also for other catalytic reactions.

## Conflict of interest

The authors declare no conflict of interest.

## Supporting information

As a service to our authors and readers, this journal provides supporting information supplied by the authors. Such materials are peer reviewed and may be re‐organized for online delivery, but are not copy‐edited or typeset. Technical support issues arising from supporting information (other than missing files) should be addressed to the authors.

Supplementary

## References

[1] T. R. Cook , D. K. Dogutan , S. Y. Reece , Y. Surendranath , T. S. Teets , D. G. Nocera , Chem. Rev. 2010, 110, 6474–6502. 21062098 10.1021/cr100246c

[2] B. Zhang , L. Wang , Y. Zhang , Y. Ding , Y. Bi , Angew. Chem. Int. Ed. 2018, 57, 2248–2252; 10.1002/anie.201712499 29333765

[3] M. Wang , Y. Yang , J. Shen , J. Jiang , L. Sun , Sustainable Energy Fuels 2017, 1, 1641–1663.

[4] S. Anantharaj , S. R. Ede , K. Sakthikumar , K. Karthick , S. Mishra , S. Kundu , ACS Catal. 2016, 6, 8069–8097.

[5] Y. Park , K. J. McDonald , K.-S. Choi , Chem. Soc. Rev. 2013, 42, 2321–2337. 23092995 10.1039/c2cs35260e

[6] X. Chang , T. Wang , P. Zhang , J. Zhang , A. Li , J. Gong , J. Am. Chem. Soc. 2015, 137, 8356–8359. 26091246 10.1021/jacs.5b04186

[7] T. W. Kim , K.-S. Choi , Science 2014, 343, 990–994. 24526312 10.1126/science.1246913

[8] F. Yu , F. Li , T. Yao , J. Du , Y. Liang , Y. Wang , H. Han , L. Sun , ACS Catal. 2017, 7, 1868–1874.

[9] S. K. Pilli , T. E. Furtak , L. D. Brown , T. G. Deutsch , J. A. Turner , A. M. Herring , Energy Environ. Sci. 2011, 4, 5028–5034.

[10] S. K. Choi , W. Choi , H. Park , Phys. Chem. Chem. Phys. 2013, 15, 6499–6507. 23529529 10.1039/c3cp00073g

[11] K. Dang , X. Chang , T. Wang , J. Gong , Nanoscale 2017, 9, 16133–16137. 29048443 10.1039/c7nr06636h

[12] Y. Wang , F. Li , X. Zhou , F. Yu , J. Du , L. Bai , L. Sun , Angew. Chem. Int. Ed. 2017, 56, 6911–6915; 10.1002/anie.201703039 28474835

[13] S. Ye , C. Ding , R. Chen , F. Fan , P. Fu , H. Yin , X. Wang , Z. Wang , P. Du , C. Li , J. Am. Chem. Soc. 2018, 140, 3250–3256. 29338218 10.1021/jacs.7b10662

[14] X.-L. Ni , X. Xiao , H. Cong , L.-L. Liang , K. Cheng , X.-J. Cheng , N.-N. Ji , Q.-J. Zhu , S.-F. Xue , Z. Tao , Chem. Soc. Rev. 2013, 42, 9480–9508. 24048328 10.1039/c3cs60261c

[15] T. V. Mitkina , N. F. Zakharchuk , D. Y. Naumov , O. A. Gerasko , D. Fenske , V. P. Fedin , Inorg. Chem. 2008, 47, 6748–6755. 18588285 10.1021/ic8003036

[16] M. Freitag , E. Galoppini , Langmuir 2010, 26, 8262–8269. 20112940 10.1021/la904671w

[17] M. Porel , A. Klimczak , M. Freitag , E. Galoppini , V. Ramamurthy , Langmuir 2012, 28, 3355–3359. 22303867 10.1021/la300053r

[18] J. Lagona , P. Mukhopadhyay , S. Chakrabarti , L. Isaacs , Angew. Chem. Int. Ed. 2005, 44, 4844–4870; 10.1002/anie.200460675 16052668

[19] D. K. Lee , K.-S. Choi , Nat. Energy 2018, 3, 53–60.

[20] H. You , D. Wu , Z.-n. Chen , F. Sun , H. Zhang , Z. Chen , M. Cao , W. Zhuang , R. Cao , ACS Energy Lett. 2019, 4, 1301–1307.

[21] H. Woo , E. Kim , J.-H. Kim , S.-W. Yun , J. C. Park , Y.-T. Kim , K. H. Park , Sci. Rep. 2017, 7, 3851. 28634386 10.1038/s41598-017-04211-9 PMC5478655

[22] P. T. Babar , A. C. Lokhande , B. S. Pawar , M. G. Gang , E. Jo , C. Go , M. P. Suryawanshi , S. M. Pawar , J. H. Kim , Appl. Surf. Sci. 2018, 427, 253–259.

[23] D. Zhao , Z. Chen , W. Yang , S. Liu , X. Zhang , Y. Yu , W.-C. Cheong , L. Zheng , F. Ren , G. Ying , X. Cao , D. Wang , Q. Peng , G. Wang , C. Chen , J. Am. Chem. Soc. 2019, 141, 4086–4093. 30699294 10.1021/jacs.8b13579

[24] L. Tong , M. Gothelid , L. Sun , Chem. Commun. 2012, 48, 10025–10027. 10.1039/c2cc35379b 22945420

[25] J. Wang , L. Gan , W. Zhang , Y. Peng , H. Yu , Q. Yan , X. Xia , X. Wang , Sci. Adv. 2018, 4 eaap7970. 29536041 10.1126/sciadv.aap7970 PMC5844707

[26] T. W. Kim , Y. Ping , G. A. Galli , K.-S. Choi , Nat. Commun. 2015, 6, 8769. 26498984 10.1038/ncomms9769 PMC4640143

[27] J. K. Kim , Y. Cho , M. J. Jeong , B. Levy-Wendt , D. Shin , Y. Yi , D. H. Wang , X. Zheng , J. H. Park , ChemSusChem 2018, 11, 933–940. 29274301 10.1002/cssc.201702173

[28] F. Li , K. Fan , L. Wang , Q. Daniel , L. Duan , L. Sun , ACS Catal. 2015, 5, 3786–3790.

[29] W. Li , F. Li , H. Yang , X. Wu , P. Zhang , Y. Shan , L. Sun , Nat. Commun. 2019, 10, 5074. 31699987 10.1038/s41467-019-13052-1 PMC6838099

[30] R. Liu , Z. Zheng , J. Spurgeon , X. Yang , Energy Environ. Sci. 2014, 7, 2504–2517.

[31] C. Zachäus , F. F. Abdi , L. M. Peter , R. van de Krol , Chem. Sci. 2017, 8, 3712–3719. 28580106 10.1039/c7sc00363c PMC5437485

[32] Y. Ma , A. Kafizas , S. R. Pendlebury , F. Le Formal , J. R. Durrant , Adv. Funct. Mater. 2016, 26, 4951–4960.

[33] J. Zhang , R. García-Rodríguez , P. Cameron , S. Eslava , Energy Environ. Sci. 2018, 11, 2972–2984.

[34] J. Xie , C. Guo , P. Yang , X. Wang , D. Liu , C. M. Li , Nano Energy 2017, 31, 28–36.

[35] Y. Liu , Y. Jiang , F. Li , F. Yu , W. Jiang , L. Xia , J. Mater. Chem. A 2018, 6, 10761–10768.

[36] B. Gao , T. Wang , X. Fan , H. Gong , P. Li , Y. Feng , X. Huang , J. He , J. Ye , J. Mater. Chem. A 2019, 7, 278–288.

[37] F. Li , Y. Li , Q. Zhuo , D. Zhou , Y. Zhao , Z. Zhao , X. Wu , Y. Shan , L. Sun , ACS Appl. Mater. Interfaces 2020, 12, 11479–11488. 32056436 10.1021/acsami.9b19418

