# Supplementary material for: A Cobalt@Cucurbit[5]uril Complex as a Highly Efficient Supramolecular Catalyst for Electrochemical and Photoelectrochemical Water Splitting
Source: Angew Chem Int Ed Engl. 2020 Nov 24;60(4):1976–85. doi: 10.1002/anie.202011069 (PMC7894348; doi:10.1002/anie.202011069)
Supplement: Supplementary file 1 — Supplementary [file ANIE-60-1976-s001.pdf]

Angewandte Chemie
Eine Zeitschrift der Gesellschaft Deutscher Chemiker

Supporting Information

A Cobalt@Cucurbit[5]uril Complex as a Highly Efficient Supramolecular Catalyst for Electrochemical and Photoelectrochemical Water Splitting

Fusheng Li[+], Hao Yang[+], Qiming Zhuo, Dinghua Zhou, Xiujuan Wu, PeiLi Zhang, Zhaoyang Yao, and Licheng Sun**

anie_202011069_sm_miscellaneous_information.pdf

Supporting information

Experimental section

Physical characterizations

Mass spectrum of Co@CB[5] was obtained using electrospray ionization-mass spectrometry (Q-TOF Micro, Micromass UK). The infrared spectra of the fabricated films were characterized by FTIR spectrometer (Nicolet Is50; Thermal Fisher Scientific 6700) with a resolution of 0.09 cm-1, the spectra of ITO-based electrodes were acquired using attenuated total reflection (ATR) mode, BiVO4-based electrodes were obtained using transmission mode by scratching off the BiVO4-based films. The secondary ion images were acquired using time of flight secondary ion mass spectra (TOF-SIMS) (nano TOF II, ULVAC-PHI) with Bi3+ as the primary ion source. The morphology and composition of the fabricated films were characterized by field emission scanning electron microscopy (FE- SEM, HITACHI UHR FE-SEM SU8220, operated at 5 kV) equipped with energy dispersive X-ray (EDX) microanalysis (Oxford EDS Inca Energy Coater 300, operated at 20 kV). High-resolution TEM (HR-TEM), high-angle annular dark-field scanning transmission electron

microscopy (HAADF-STEM) and energy dispersive spectroscopy (EDS) elemental maps were acquired using a FEI Talos F200X instrument at an acceleration voltage of 200 kV. The surface composition of the electrode films was investigated using X-ray photoelectron spectroscopy (XPS) on a Thermo Scientific™ ESCALAB™ Xi$^+$. The concentration of Co was measured using Inductively Coupled Plasma Optical Emission Spectroscopy (ICP-OES) with a Thermo Scientific iCAP 6000 series instrument. The samples on electrodes were digested in 2mL aqua regia with ultrasonic in closed vessel and then diluted 10 times as the sample solution. Co standard solutions were measured prior to the samples to calibrate and obtain the standard curve. Co concentrations were determined using four different wavelengths: 231.1 nm, 235.3 nm, 237.8 nm and 238.8 nm. The average concentrations obtained at different wavelengths were taken for data evaluation.

Materials and reagents

Bismuth nitrate pentahydrate ($Bi(NO_3)_3·5H_2O$, 99.0 %), vanadyl acetylacetonate ($VO(acac)_2$, 99 %), lactic acid (90 %), HNO_3 (98 %), KI (99 %) and cobalt nitrate hexahydrate ($Co(NO_3)_2·6H_2O$, 99.99 %) were purchased from Aladdin. D_2O (99.9%) was purchased from inno-chem, Inc., sodium tetraborate (anhydrous, 99.95%) was purchased from Alfa Aesar. High purity water (18.2 $MΩ·cm^{-1}$) supplied by a Milli-Q system (Millipore, Direct-Q 3 UV) was used in all experiments. Fluorine-doped tin oxide (FTO, NSG 10Ω 10 mm×25 mm×1.1 mm) substrate was purchased from local company, before using, the FTO substrate was ultrasonically cleaned in deionized water, ethanol and acetone for 15 min, respectively. All other reagents were commercially available and used as received. Organic solvents were analytical reagent grade and used without further purification.

Fabrication of porous ITO substrate

Porous ITO films were prepared by doctor-blading the paste onto ITO conducting glass using Scotch tape as a spacer, followed by sintering in an oven at 450℃ for 30 min. The paste was prepared by ball milling a mixture of indium tin oxide nanoparticles (1 g), ethyl cellulose (150 mg), ethanol (8 mL), terpineol (4 mL) and acetylacetone (100 μL) for 4h.

Fabrication of $BiVO_4$ films

$BiVO_4$ films were prepared by the modification of a literature procedure.[1] Briefly,

$Bi(NO_3)_3 \cdot 5H_2O$ (0.3675 g, 0.75 mmol) was added to 50 mL water solution containing 136 μL lactic acid. After ultrasonication for 5 min, KI (3.354g, 20 mmol) was carefully added to the solution and the mixture was vigorously stirred for 30 min. This solution was slowly mixed with 20 mL ethanol containing p-benzoquinone (0.1g, 0.92 mmol), and vigorously stirred for 10 minutes. The final pH was adjusted to 3.4 by adding HNO_3 to form the BiOI platting solution. A typical three-electrode cell containing a FTO working electrode, a saturated Ag/AgCl reference electrode (0.194 V vs. NHE) as the reference electrode and a Pt mesh counter electrode were used for electrodeposition. The cathodic deposition was performed potentiostatically at −0.35 V vs. Ag/AgCl for 20 s, following −0.10 V vs. Ag/AgCl for a few minutes, equivalent to passing a total charge of 0.37 C cm^{-2}.

The BiOI film was converted to $BiVO_4$ by a thermal treatment in air at 450 °C for 2 h after covering the BiOI film with 40 μL of a DMSO solution containing 0.2 M $VO(acac)_2$ (ramping rate 2 °C/min). After cooling to room temperature, the as-annealed electrodes were soaked in a 1 M NaOH solution for 30 min to remove the V_2O_5 from the surface of $BiVO_4$. The resulting film was then rinsed with deionized water and dried with air stream.

Fabrication of Co@CB[5] functionalized electrode

For the preparation of Co@CB[5]/ITO electrode, first, the porous ITO substrate was immersed in a CB[5] solution (1.0 mM) for 8 hours (denoted as CB[5]/ITO). After washing with deionized water, the CB[5]/ITO film was immersed in a $Co(NO_3)_2$ solution (1.0 mM) for another 8 hours, resulting in the generation of Co@CB[5]/ITO electrode. Please note that the pH of $Co(NO_3)_2$ solution was 5.6 while the precipitation pH of Co^{2+} was higher than 7 at room temperature, thus, Co^{2+} was hard to hydrolyze and form precipitation in the $Co(NO_3)_2$ solution under such experimental conditions.

For the preparation of Co@CB[5]/$BiVO_4$ electrode, the porous $BiVO_4$ substrate was immersed in a CB[5] solution (1.0 mM) for 20 minutes (denoted as CB[5]/$BiVO_4$). After washing with deionized water, the CB[5]/$BiVO_4$ film was immersed in a $Co(NO_3)_2$ solution (1.0 mM) for another 20 minutes, resulting in the generation of Co@CB[5]/$BiVO_4$ electrode.

Electrochemical measurements

Cyclic voltammetry (CV) measurements were carried out on a CHI 760e potentiostat

(Shanghai Chenhua Instrument Co., LTD). All electrochemical tests were performed at 25 °C in a similar three-electrode system which was used as described above, 1.0 M borate buffer solution (pH 9.2) working as the electrolyte to evaluate the catalytic activities of the electrode films. J-V curves were obtained by cyclic voltammetry (CV) at a scan rate of 50 mV s^{-1} with *iR* compensation. The recorded potential was converted to RHE using the Nernst equation ($E_{RHE} = E_{Ag/AgCl} + 0.194 + 0.059$ pH).

Calculation of turn over frequency (TOF)

The loading of Co^{2+} cation Γ (mol cm^{-2}) has a linear relationship with the peak current i_p (here is the redox peak of $Co^{II/III}$) given by ***eqn. S1***, where ***n*** is number of electrons (for $Co^{III/II}$ is ***n*** = 1), ***v*** is scan rate (V s^{-1}), ***A*** is surface area (cm^2), ***F*** is Faraday's constant (96485 C mol^{-1}), ***R*** is ideal gas constant (8.314 $JK^{-1}mol^{-1}$), ***T*** is temperature (298 K).[2]

$$i_{p(Co^{III/II})} = \frac{n^2F^2\upsilon A\Gamma}{4RT} \qquad eqn.\ S1$$

The loading of Co^{2+} cation on the surface of FTO was estimated according to the linear relationship between the peak current of $Co^{2+/3+}$ and the scan rate (***eqn. S2***).

$$Slope = \frac{n^2F^2A\Gamma}{4RT} \qquad eqn.\ S2$$

The TOF of Co@CB[5]/FTO was calculated by ***eqn.S3***, where ***J*** is the OER current density, which was achieved from CV under a low scan rate; ***A*** is the surface area of the electrode; ***F*** is the Faraday constant; and Γ is the amount of electroactive Co^{2+} cation obtained from ***eqn.S2.***[3]

$$TOF = \frac{JA}{4F\Gamma A} \qquad eqn.\ S3$$

Photoelectrochemical measurements

All photoelectrochemical measurements were carried out at room temperature by using a CHI 760E electrochemical analyzer (Shanghai Chenhua Instrument Co., LTD). The photoelectrochemical performances of photoanodes were measured with a three-electrode configuration. The simulated solar illumination was obtained by passing light from a 300 W Xenon arc lamp (EXCELITAS, PE300BFA) equipped with an AM 1.5G filter, the power

intensity of the incident light was calibrated to 100 mW cm^{-2} by a Newport OMM-6810B photometer (OMH-6742B, Silicon detector, 350-1100nm). J-V curves were obtained by linear sweeping voltammetry (LSV) at a scan rate of 10 mV s^{-1}. Intensity Modulated Photocurrent Spectroscopy (IMPS) spectra were recorded by a Zahner IMPS electrochemical workstation. Intensity-modulated light was provided by a white light-emitting diode (LED) with light intensity of 100 mW cm^{-2} that allowed superimposition of sinusoidal modulation (~10%) on a dc illumination level. The modulation amplitude of lamp voltage was 2 mV. The IMPS data was recorded at different applied potentials over the 1 kHz – 50 mHz frequency range.

The applied bias photon-to-current efficiency (ABPE) was calculated from the J-V curves under illumination using the ***eqn. S4***:

$$ABPE(\%) = \frac{(1.23 - V_{RHE}) \times \left(J_{light} - J_{dark}\right)}{P_{light}} \times 100\% \qquad eqn.\ S4$$

Where V_{RHE} is the applied potential versus RHE and P_{light} (100 mW/cm^2) is the power density of AM 1.5G.

The incident photon to current efficiency (IPCE) of each wavelength was determined using the illumination from a 300 W Xe arc lamp. The monochromatic light was produced using a monochromator. The light intensity (P_λ) at each wavelength (λ) was determined by Newport OMM-6810B photometer (OMH-6742B, Silicon detector, 350-1100nm), J_{light} and J_{dark} are the measured photocurrent and dark current respectively, and the IPCE values were calculated using the ***eqn. S5***.

$$IPCE(\%) = \frac{1240 \times \left(J_{light} - J_{dark}\right)}{\lambda \times P_\lambda} \times 100\% \qquad eqn.\ S5$$

Calculation of the surface charge separation efficiency ($\eta_{surface}$)

The surface charge separation efficiency ($\eta_{surface}$) was calculated using the ***eqn. S6***.

$$\eta_{surface} = \frac{J_{water}}{J_{sulfite}} \times 100\% \qquad eqn.\ S6$$

Where J_{water} is the current density of water oxidation at a certain applied potential, $J_{sulfite}$ is the current density of sodium sulfite as a hole scavenger.

KIEs experiments

An anhydrous sodium tetraborate H_2O solution (0.1 M) was used for KIE measurements, the pH value of the solution (H_2O) was measured to be 9.3. For studies in deuterium solutions, the deuterated solution was prepared by adding anhydrous sodium tetraborate into D_2O (0.1 M), the pH value of the borate buffer (D_2O) was measured to be 9.4. Other testing conditions of photoelectrochemical/electrochemical KIEs measurements were the same with that of conditions mentioned above. The corresponding current densities at a certain overpotential η were abbreviated as j_{H2O} and j_{D2O}. Then, the $KIEs_{(H/D)}$ was defined as ***eqn. S7***.

$$KIEs_{(H/D)} = \left[\frac{k_{H_2O}}{k_{D_2O}}\right]_{\eta} = \left[\frac{j_{H_2O}}{j_{D_2O}}\right]_{\eta} \qquad eqn. S7$$

For an electrochemical reaction, according to Butler–Volmer equation, the activation overpotential is the potential difference above the equilibrium value required to produce a current, which depends on the activation energy of the redox event.[2] Because of that the catalytic activity will raise with the overpotential adding on the catalyst increasing, thence, for $KIEs_{(H/D)}$ measurements, the current densities j_{H2O} and j_{D2O} should be contrasted at the same overpotential.

The overpotential can be corrected by ***eqn. S8*** for the measurement in aqueous solution.

$$\eta^{H_2O} = E_{Ag/AgCl}^{read\ in\ H} + 0.059pH + E_{Ag/AgCl}^{H} - 1.229\,V_{RHE} \qquad eqn.\ S8$$

Where $E_{Ag/AgCl}^{read\ in\ H}$ is the potential read by using the Ag/AgCl as reference electrode in H_2O solution; $E_{Ag/AgCl}^{H}$ is the equilibrium potential for Ag/AgCl couple in the NHE scales (0.197 V).[2, 4] The pH of 100 mM sodium tetraborate (anhydrous) H_2O solution measured by pH meter was 9.3.

As the equilibrium potentials for the D_2/D^+ reaction, and the O_2/D_2O electrochemical reactions are different from their equivalent reactions in H_2O, overpotentials should be corrected to the RDE (reversible "deuterium" electrode in D_2O) scales before being used to calculate the KIEs.[5] The different free energy of formation ($\Delta_{formation}$ G) of H_2O (−237.18 kJ/mol) vs D_2O (−243.49 kJ/mol) leads to different equilibrium potentials of 1.229 V_{RHE} and

1.262 V_{RDE} for water oxidation.[5-6] The overpotential can be corrected by ***eqn. S9*** for the measurement in D_2O solution.

$$\eta^{D_2O} = E_{Ag/AgCl}^{read\ in\ D} + 0.059pD + E_{Ag/AgCl}^{D} - 1.262\ \mathrm{V}_{RDE} \qquad eqn.\ S9$$

Where $E_{Ag/AgCl}^{read\ in\ D}$ is the potential read by using Ag/AgCl as reference electrode in D_2O solution, $E_{Ag/AgCl}^{D}$ is the equilibrium potential for Ag/AgCl couple in the NDE scales (normal "deuterium" electrode). The equilibrium potential for the deuterium couple (D_2/D^+) is different from that of the proton couple (H_2/H^+) (−0.013 V),[6] which means the difference of NDE and NHE scales is −0.013 V, then the equilibrium potential difference for Ag/AgCl couple in NDE and NHE scales ($E_{Ag/AgCl}^{D}$ - $E_{Ag/AgCl}^{H}$) is −0.013 V, then $E_{Ag/AgCl}^{D}$ is 0.184 V.

Because of that 100 mM sodium tetraborate (anhydrous) H_2O solution and D_2O solution were used as electrolytes, For estimating pD, measurements were conducted in deuterated solvents using the pH meter and the pD was calculated by the following equation: pD = $pH^{meter\ reading}$+0.40.[7] Then, the ***eqn. S9*** become:

$$\eta^{D_2O} = E_{Ag/AgCl}^{read\ in\ D} + 0.059(pH + 0.40) + E_{Ag/AgCl}^{D} - 1.262\ \mathrm{V}_{RDE} \quad eqn.\ S10$$

The pH of 100 mM sodium tetraborate (anhydrous) D_2O solution was measured as 9.4 in our experiments. Then the overpotential was corrected by ***eqn. S10*** for the measurement in D_2O solution.

Determination of Faradaic efficiency

The amounts of oxygen and hydrogen evolution were determined by gas chromatography (Techcomp GC 7890T, Ar carrier gas, Thermo Conductivity Detector). The Faradaic efficiency was calculated according to the integrated charge passed and the amount of O_2 evolved.

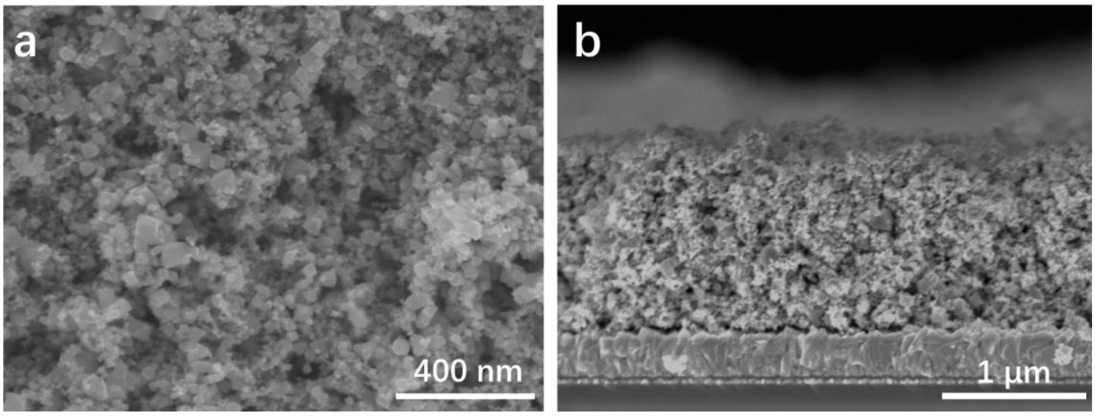

Figure S1. SEM images of the porous ITO substrate. (a) top view, (b) cross-sectional view.

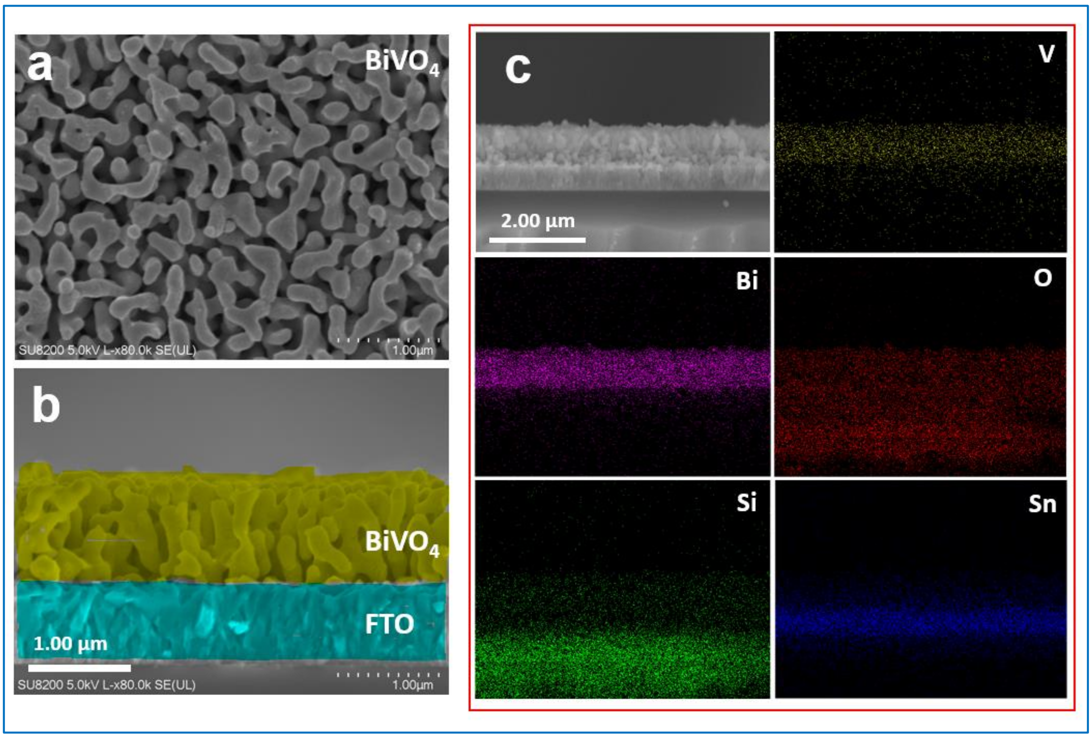

Figure S2. SEM images of the porous $BiVO_4$ photoanode by (a) top view, (b) side view and (c) EDS mapping images of porous $BiVO_4$ photoanode by side view.

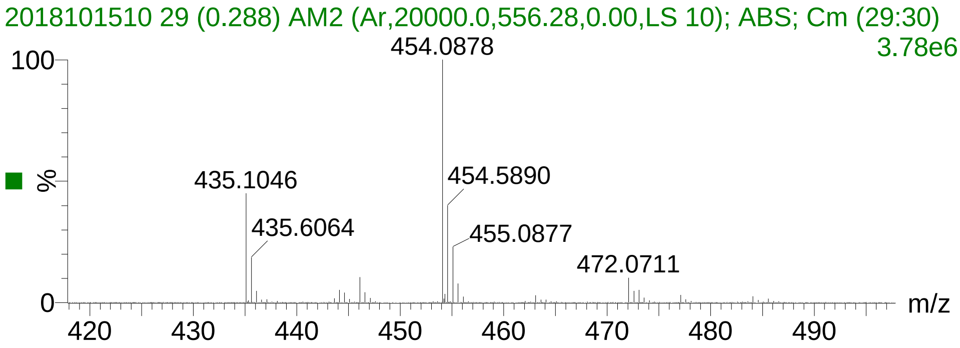

Figure S3. Electrospray mass spectrum of the solution of Co@CB[5] host-guest complex (1.0 mM) prepared by mixing CB[5] and Co(NO3)2 in a molar ratio of 1:1.

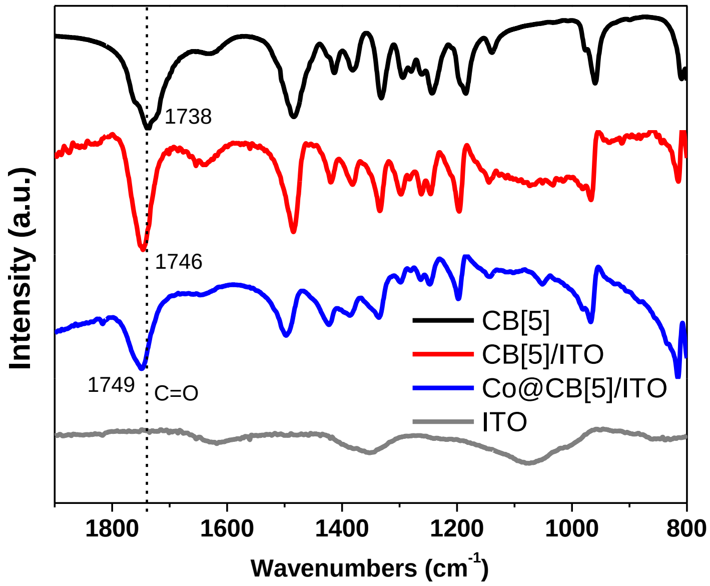

Figure S4. Attenuated total reflection infrared (ATR-IR) spectra of ITO, Co@CB[5]/ITO, CB[5]/ITO electrode and bare CB[5] powder.

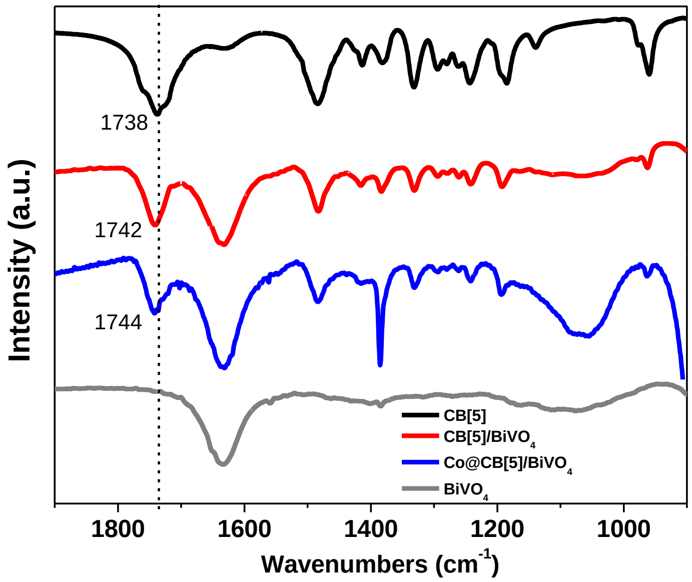

Figure S5. Fourier-transform infrared spectra of BiVO4, Co@CB[5]/BiVO4 Co@CB[5]/BiVO4, CB[5]/ITO and bare CB[5] powder.

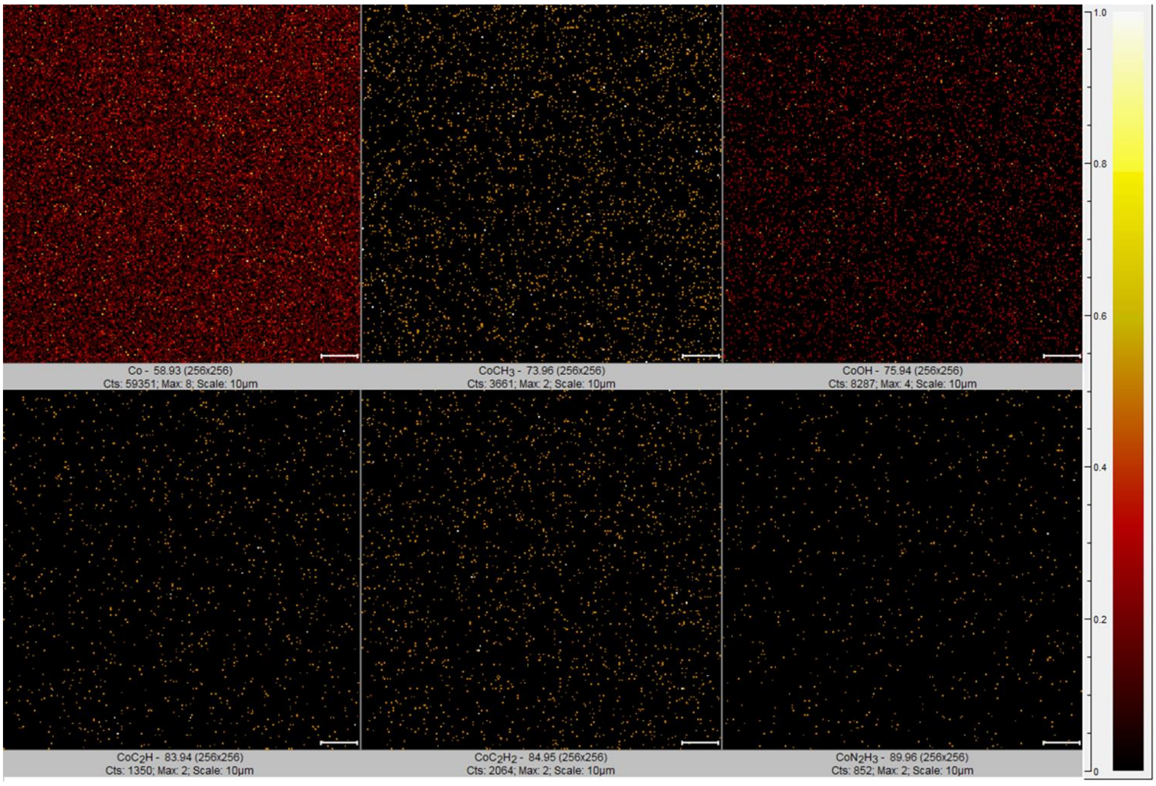

Figure S6. Time-of-flight secondary ion mass (TOF-SIMS) images of Co@CB[5]/ITO.

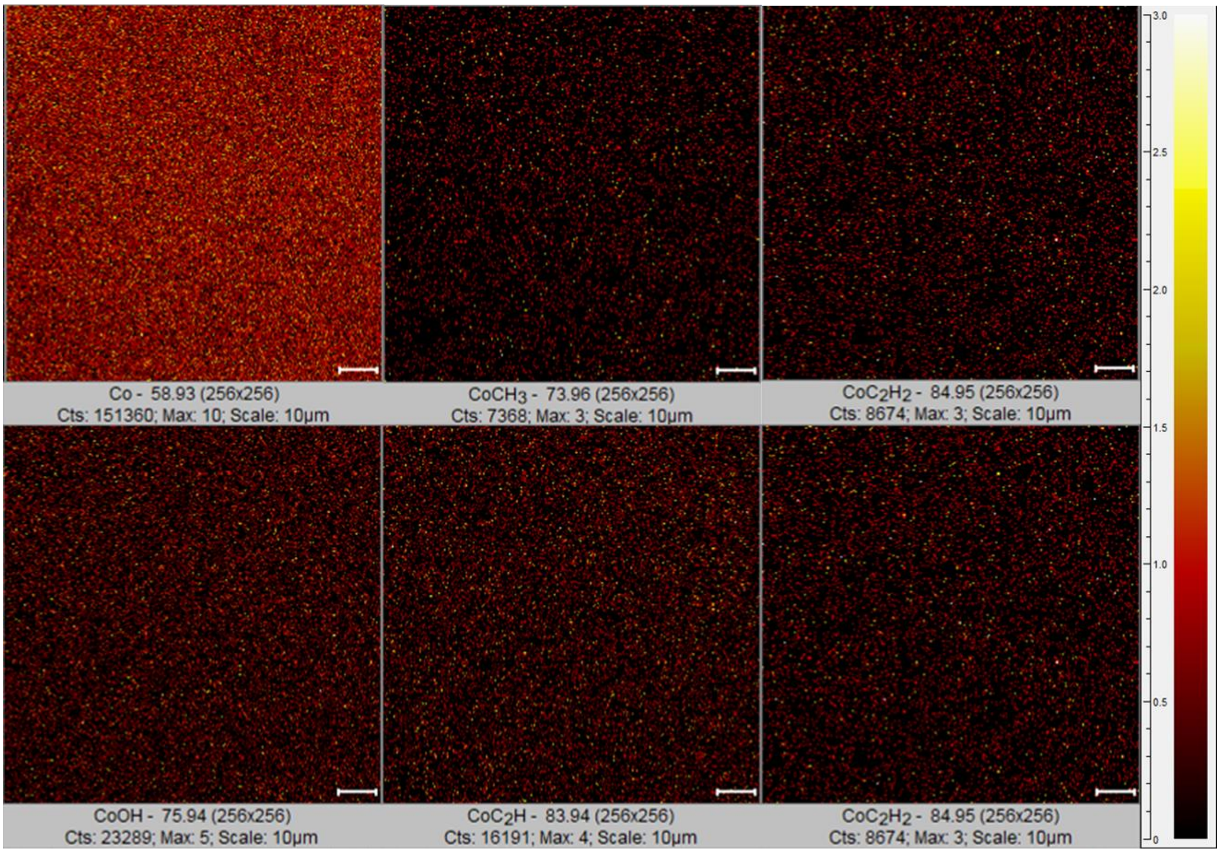

Figure S7. TOF-SIMS images of Co@CB[5]/$BiVO_4$

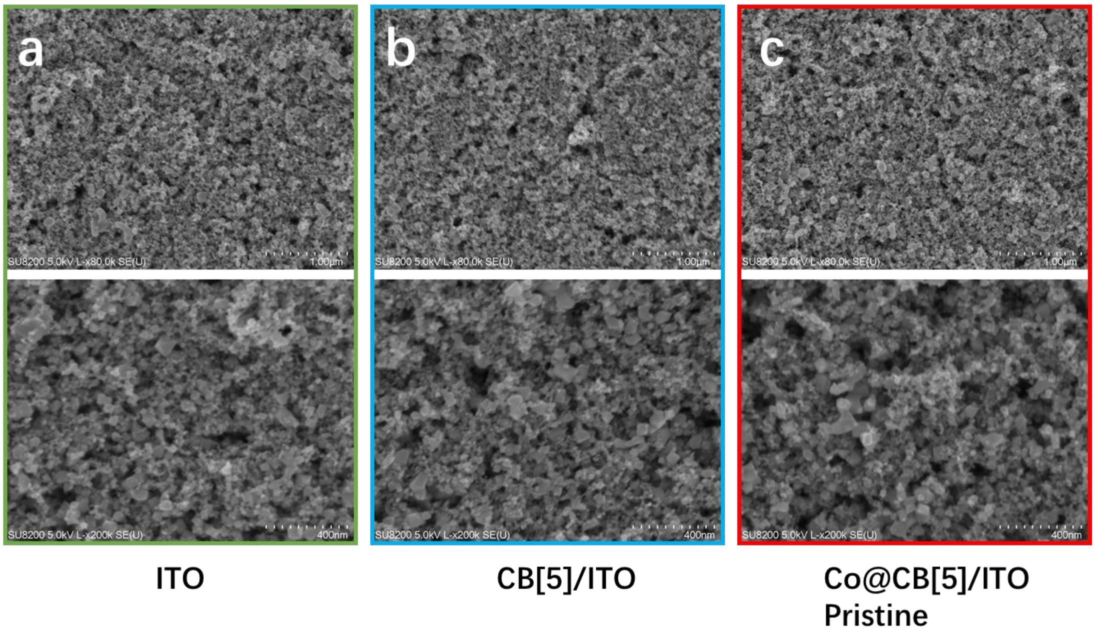

Figure S8. SEM images of the prepared electrodes. (a) ITO substrate, (b) CB[5]/ITO, (c) Co@CB[5]/ITO.

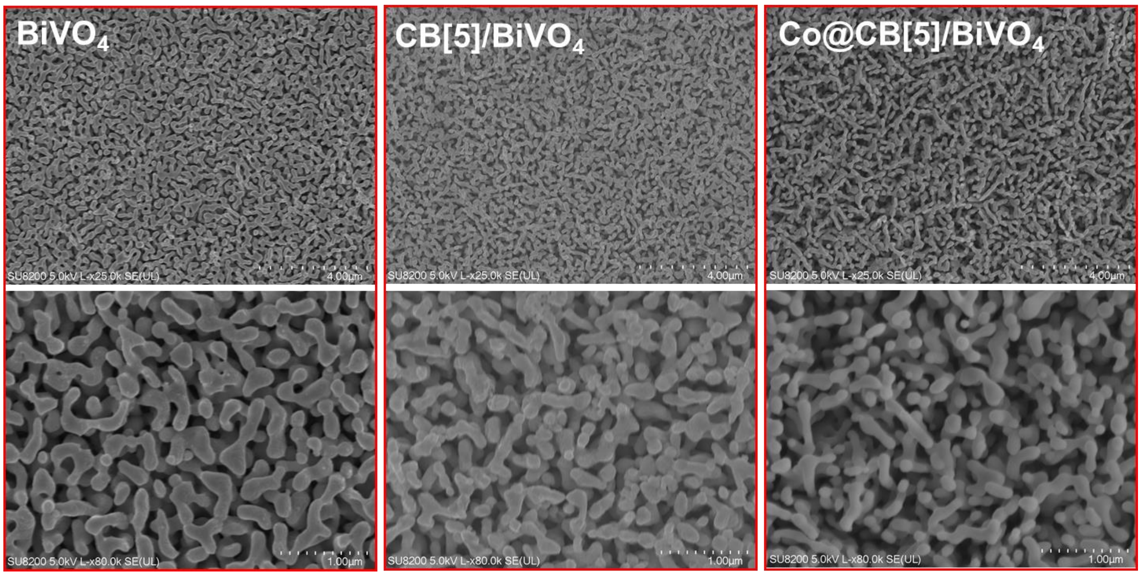

Figure S9. SEM images of the prepared porous $BiVO_4$, CB[5]/$BiVO_4$ and Co@CB[5]/$BiVO_4$ photoanodes.

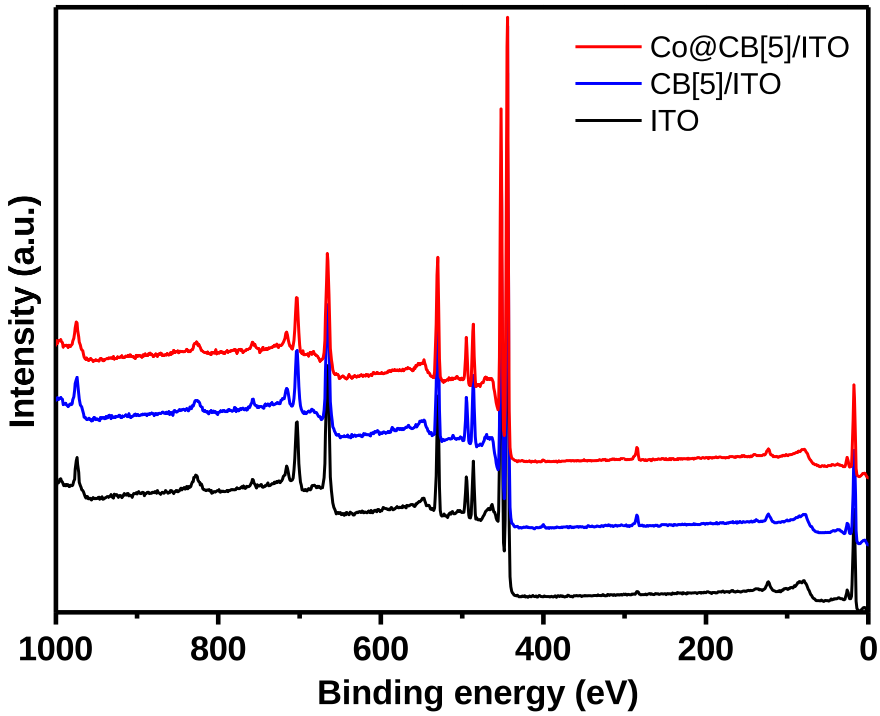

Figure S10. XPS survey spectra of ITO, CB[5]/ITO and Co@CB[5]/ITO.

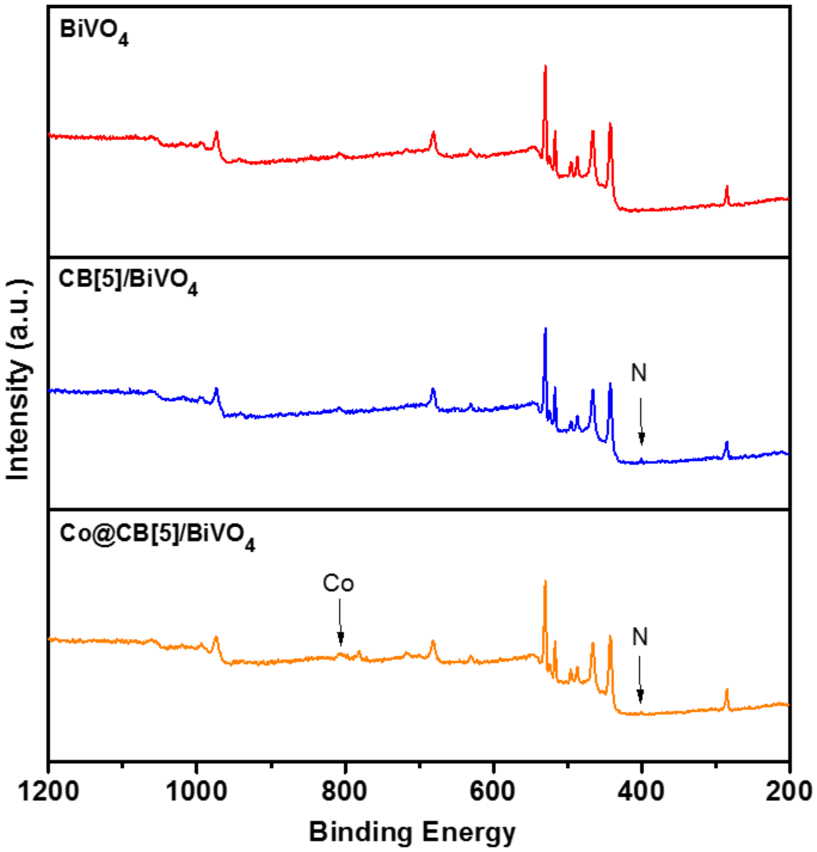

Figure S11. XPS survey spectra of $BiVO_4$, CB[5]/$BiVO_4$ and Co@CB[5]/$BiVO_4$

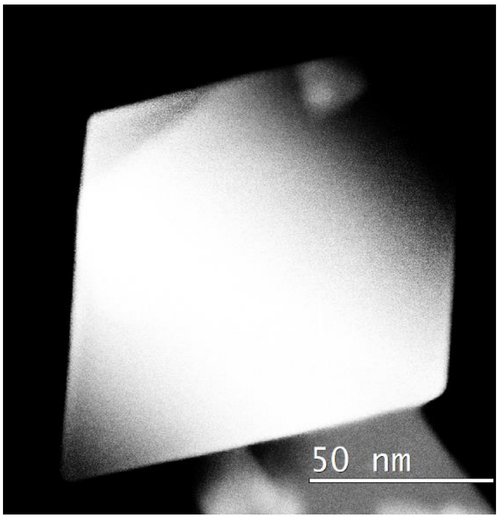

Figure S12. The HAADF image of integral Co@CB[5]/ITO particle.

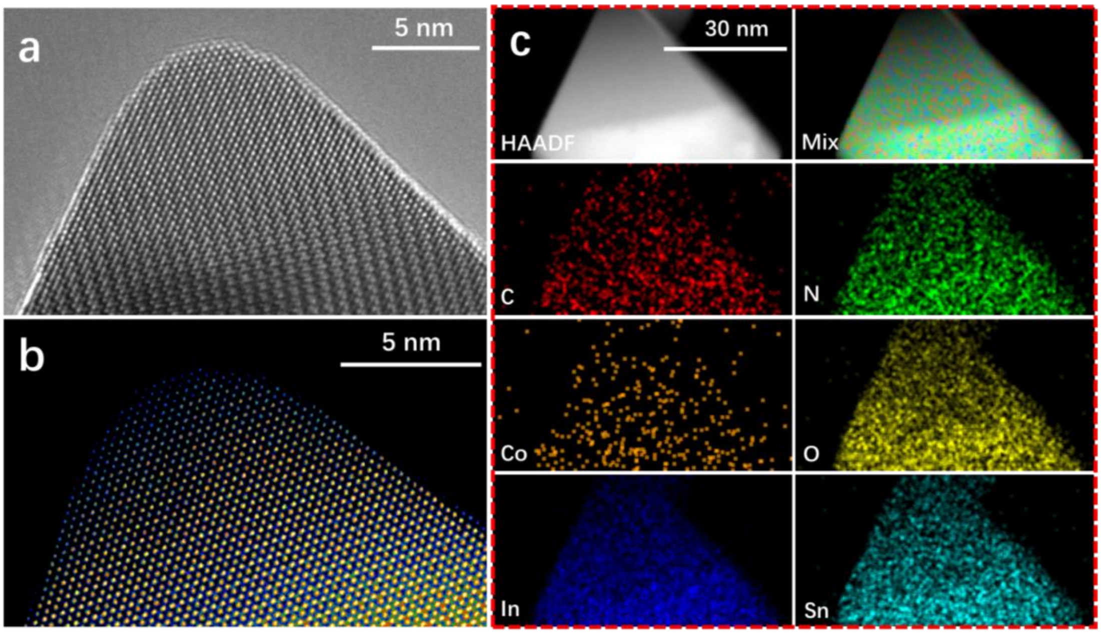

Figure S13. Atomic-resolution TEM image (a) and corresponding HAADF image (b) of Co@CB[5]/ITO particle, showing the high crystallinity of bulk ITO particle and clear particle boundary without heterojunction layer coverage. (c) Energy-dispersive X-ray spectroscopy (EDS) mappings of Co@CB[5]/ITO sample, showing the homogenous distribution of Co and N over the ITO particle.

The atomic-resolution TEM images of Co@CB[5]/ITO sample that operated in bright field showed a clear and smooth boundary of ITO nano-particle (***Fig. S13a***). TEM images operated in high-angle annular dark-field (HAADF) was applied to further observe the atomic structure of ITO surface (***Fig. S13b***). The clear and well-ordered lattice of ITO nanoparticle in atomic-scale HAADF images reveal that there is no structural decomposition observed after attachment of Co@CB[5] complex, in the meantime, no other amorphous layer or heterojunction film covered on the boundary of ITO nano-particle were observed at the film surface after the immobilization of Co@CB[5] complex. The TEM elemental mapping of Co@CB[5]/ITO nano-particle in ***Fig. S13c*** clearly verify the homogeneous distribution of Co, N and C components over the entire particle, which illustrated the uniform distribution of Co@CB[5] complex.

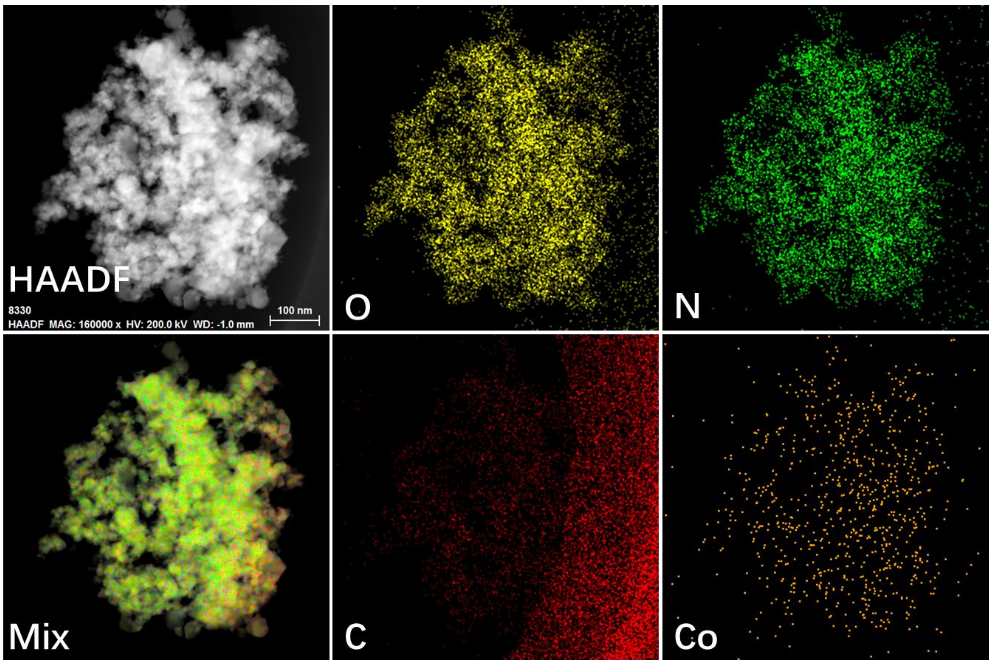

Figure S14. Large-scale EDS mapping images of Co@CB[5]/ITO sample, Co, N and C components maintain uniform distribution.

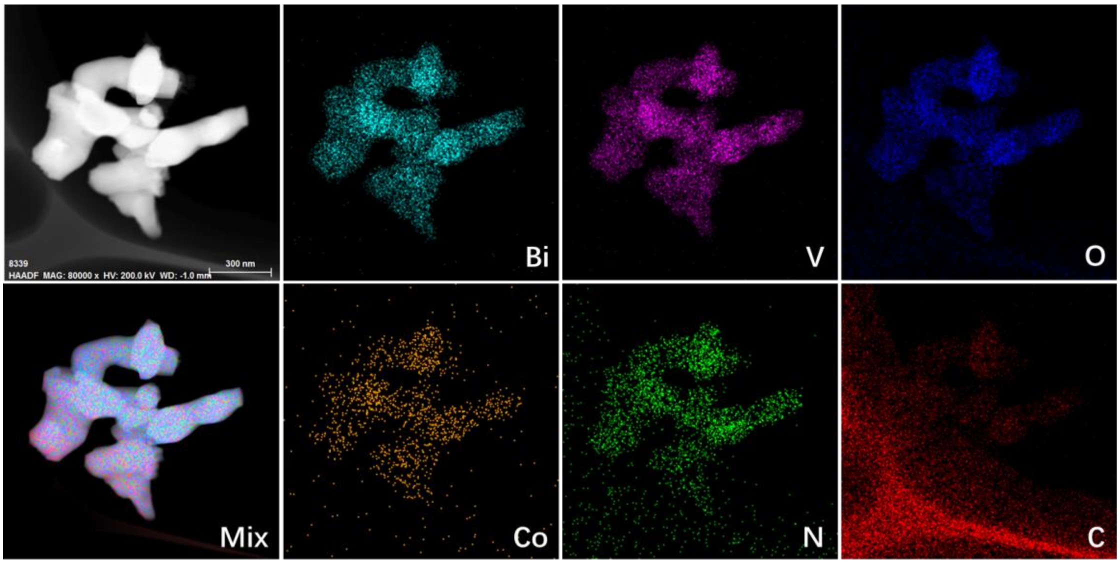

Figure S15. Large-scale EDS mapping images of Co@CB[5]/$BiVO_4$ sample. Co, N and C components maintain uniformly distribution.

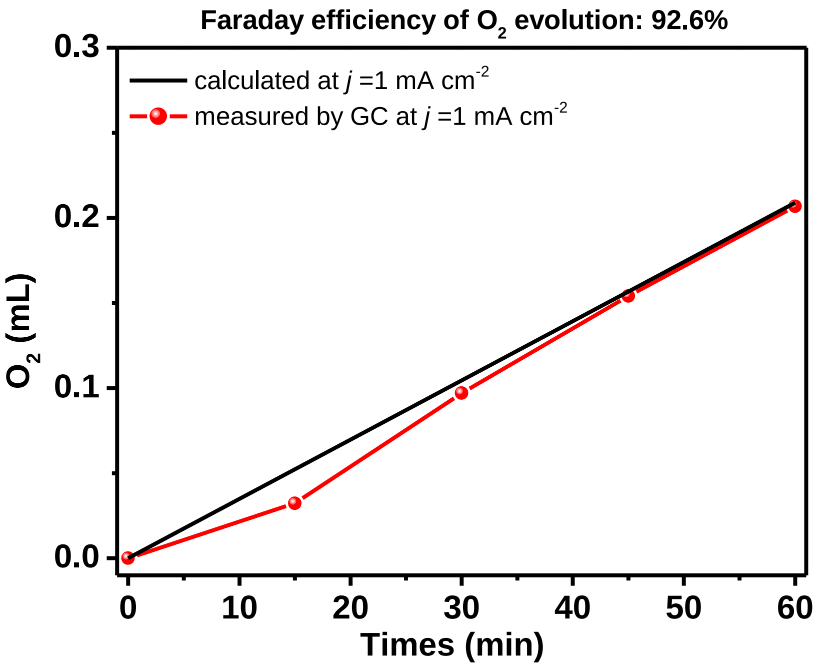

***Figure S16*.** The Faradaic efficiency of Co@CB[5]/ITO electrode for OER in 1.0 M borate buffer. The Faradaic efficiency was determined by comparing the measured amount of oxygen gas and the theoretical value calculated based on the transferred charge. Oxygen evolution detected by gas chromatography and the quantitative yields of 92.6% was obtained at j = 1 mA cm^{-2}.

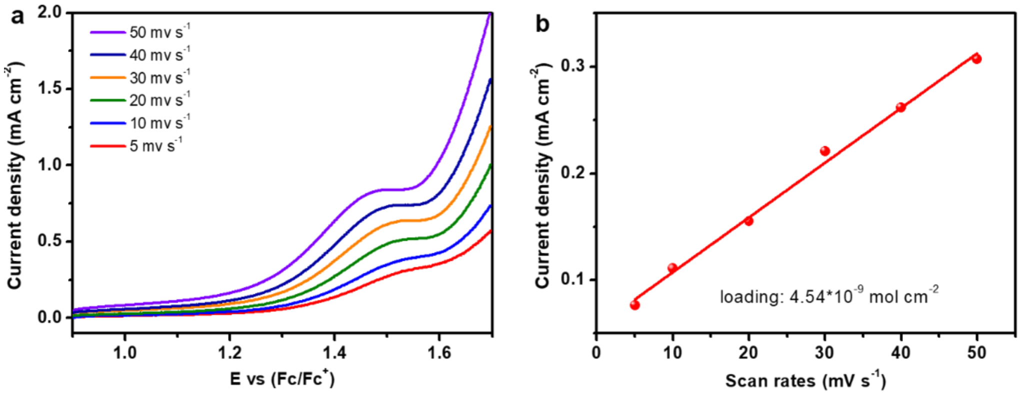

Figure S17. (a) LSV curves of Co@CB[5]/ITO electrode at different scan rates in a 0.1 M nBt_4NPF_6 acetonitrile solution at room temperature. (b) dependence of peak current density on different scan rates (the calculated loading amounts of redox activated cobalt is 4.54×10^{-9} mol cm^{-2}).

Table S1. OER performance of recently published catalysts immobilized on conducting glass substrate.

Catalyst	Catalyst immobilization method	Catalyst loading [nmol cm^{-2}]	Electrolyte	η [mV at j= 1 mA cm^{-2}]	TOF [h^{-1} at η = 530 mV]	Ref
**Co@CB[5]/ porous ITO**	**Soaking**	**4.54**	1.0 M KBi (pH 9.2)	**480**	**15120 (4.2 s^{-1})**	**This work**
Fe-based	electrodeposition	10.2	0.1 M Pi (pH 7)	480	756	Angew. Chem. Int. Ed. 2015, 54, 4870
MnCat	electrodeposition	80	0.1 M Pi (pH 7)	590	36	Energy Environ. Sci., 2012, 5, 7081
Co-based	electrodeposition	100	0.1 M Pi (pH 7)	550	61.2	Angew. Chem. Int. Ed. 2015, 54, 4870; Science 2008, 321, 1072
$[Co(IDA)_2]^{2-}$	electrodeposition	400	0.6 M NaBi (pH 9.2)	~425	N/A	ChemSusChem, 2015, 8, 1394-1403
$[Co(NTA)(OH_2)_2]^{-}$	electrodeposition	190	0.6 M NaBi (pH 9.2)	~450	N/A	ChemSusChem, 2015, 8, 1394-1403
$Mn_3(PO_4)_2$	precipitation	611	0.5 M Pi (pH 7)	680 (0.32 mA cm^{-2})	4.4 (680 mV)	J. Am. Chem. Soc. 2014, 136, 7435
$LiMnP_2O_7$	solid-state synthesis	1059	0.5 M Pi (pH 7)	680 (0.5 mA cm^{-2})	4.2 (680 mV)	J. Am. Chem. Soc. 2014, 136, 4201
NiO_x-en	electrodeposition	270	0.1 M NaBi (pH 9.2)	510	54 (610 mV)	Energy Environ. Sci. 2013, 6, 579
Cu-Bi	electrodeposition	N/A	0.2 M Bi (pH 9)	576	N/A	ACS Catal. 2015, 5, 627

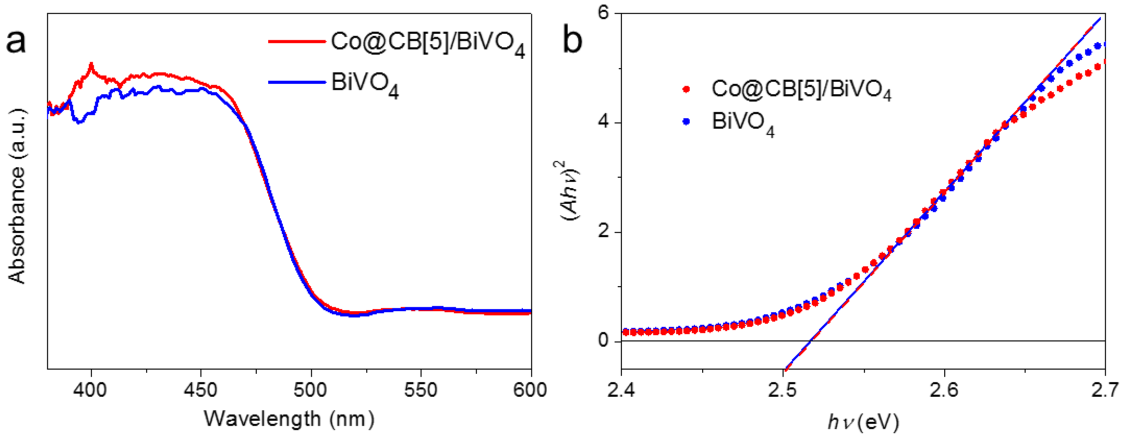

Figure S18. (a) UV-Vis diffuse spectra of $BiVO_4$ and Co@CB[5]/$BiVO_4$, (b) Tauc plots of $BiVO_4$ and Co@CB[5]/$BiVO_4$.

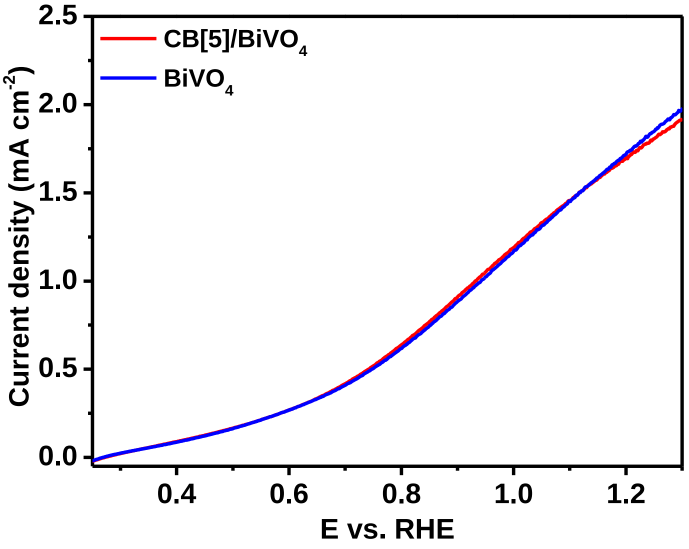

Figure S19. LSV curves of CB[5]/$BiVO_4$ and $BiVO_4$.

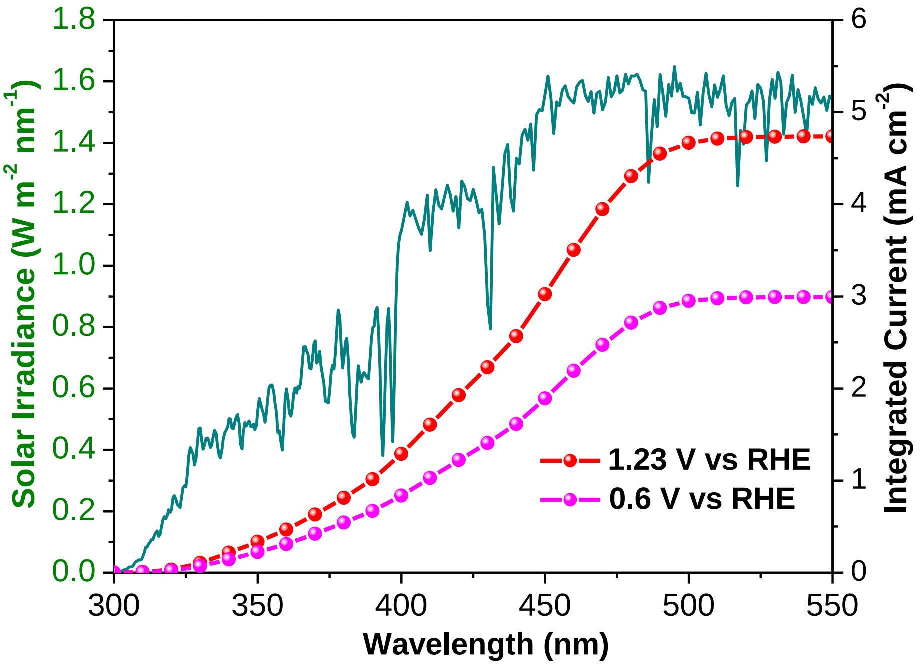

Figure S20. Solar irradiance of AM 1.5G (ASTM G173-03) and calculated photocurrent of Co@CB[5]/$BiVO_4$ electrode by integrating IPCE at 0.6 V and 1.23 V vs. RHE over the photon flux of AM 1.5G.

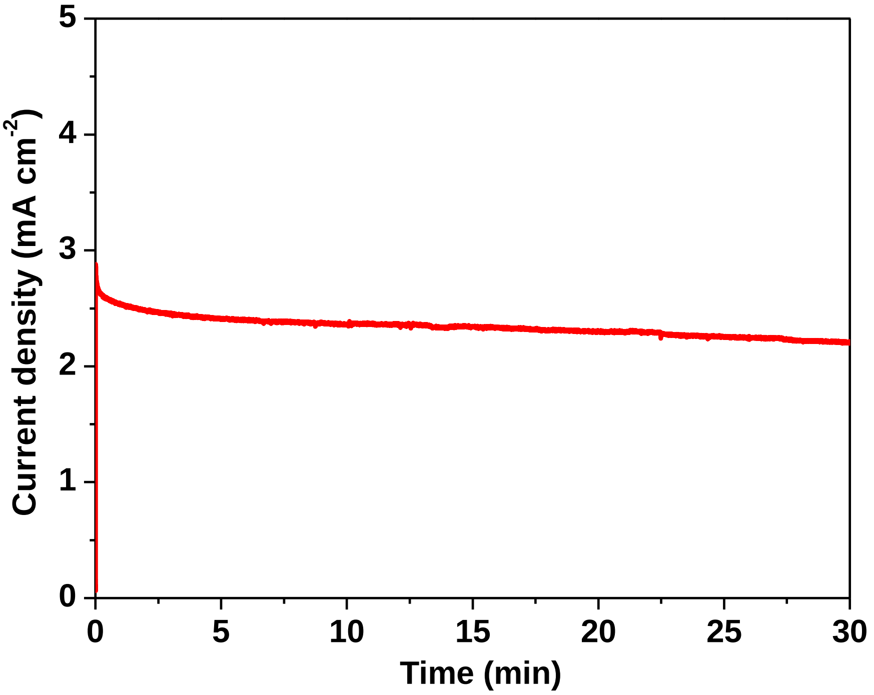

Figure S21. I-t curve of Co@CB[5]/$BiVO_4$ photoanode at a constant applied potential of 0.6 V vs. RHE. Measurement was carried out in a 1 M borate buffer (pH 9.2) under AM 1.5G simulated sunlight irradiation (100 mW cm^{-2}).

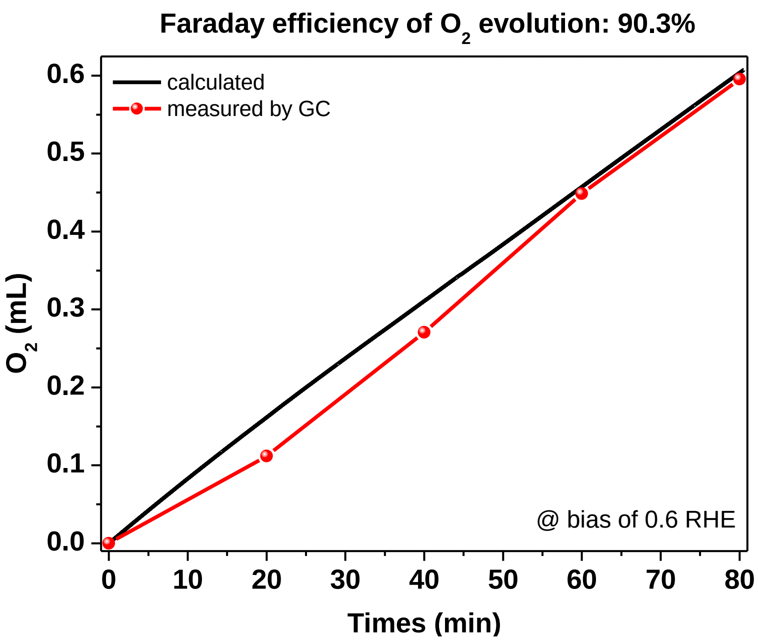

Figure S22. The Faradaic efficiency of Co@CB[5]/$BiVO_4$ photoanode for OER. Oxygen evolution detected by gas chromatography and the charge passed during the photolysis of Co@CB[5]/$BiVO_4$ photoanode at an applied potential of 0.6 V vs. RHE. Measurements were carried out in a 1 M borate buffer (pH 9.2) under AM 1.5G simulated sunlight irradiation (100 mW cm^{-2}).

Table S2. PEC performance of $BiVO_4$ based photoanodes reported in literature

Photoanode	Catalyst immobilization method	Electrolyte	Photocurrent density (1.23 V_{RHE})	Max ABPE (%)	Ref
**Co@CB[5]/$BiVO_4$**	**Soaking**	**1.0 M KBi (pH 9.2)**	**4.8 mA cm^{-2}**	**1.79**	**This work**
Co_4O_4 Cubane/ $BiVO_4$	Drop casting	0.5 M NaBi (pH 9.3)	5 mA cm^{-2}	1.8	*Angew. Chem.* 2017, 129, 7015-7019.
ultrathin CoOOH/$BiVO_4$	plasma-exfoliation	0.2 M Na_2SO_4 (pH=7)	4.9 mA cm^{-2}	0.85	*J. Mater. Chem. A 2019, 7, 4415.*
$FeCoO_x$/BiVO4	Photo-electrodeposition	1 M KBi (pH 9.5)	4.82 mA cm^{-2}	1.16	*Adv. Funct. Mater.* 2018, 1802685.
Co-Cubanes/ pGO/LDH/$BiVO_4$	Soaking	1.0 M KBi (pH 9.0)	4.45 mA cm^{-2}	2.0	*J. Am. Chem. Soc.* 2018, 140, 3250-3256.
NiOOH/FeOOH/$BiVO_4$	Photo-electrodeposition	0.5 M KPi (pH 7)	4.5 mA cm^{-2}	1.75	*Science*, 2014, 343, 990-994
NiFeOx-Bi /$BiVO_4$	Photo-electrodeposition	1 M KBi (pH 9.3)	4.3 mA cm^{-2}	2.02	*Adv. Energy Mater.* 2016, 6, 1501645.
Co-salophen@Nafion/ $BiVO_4$	Drop casting	0.1 M KPi (pH 7)	4.27 mA cm^{-2}	1.18	*J. Mater. Chem. A* 2018, 6, 10761-10768.
NiOOH/NiO/CoO_x/ $BiVO_4$	Atomic layer deposition (ALD)	0.1 M KPi (pH 7)	3.5 mA cm^{-2}	1.5	*J. Am. Chem. Soc.* 2015, 137, 5053-5060
CoMOF/$BiVO_4$	Drop casting/Soaking	0.5 M Na_2SO_4	3.1 mA cm^{-2}	0.9	*ChemSusChem,* 2018, 11(16), 2710-2716.
CoTCPP/Al_2O_3/ $BiVO_4$	Soaking	0.1 M Na_2SO_4 (pH 6.8)	2.1 mA cm^{-2}	0.45	*ACS Appl. Mater. Interfaces* 2016, 8, 18577-18583.
[(cy)Ru(L_2bpy)OH_2]$^+$/ $BiVO_4$	Soaking	0.1 M KPi (pH 7.1)	1.3 mA cm^{-2}	N/A	*J. Phys. Chem. C*, 2015, 119, 7275-7281.

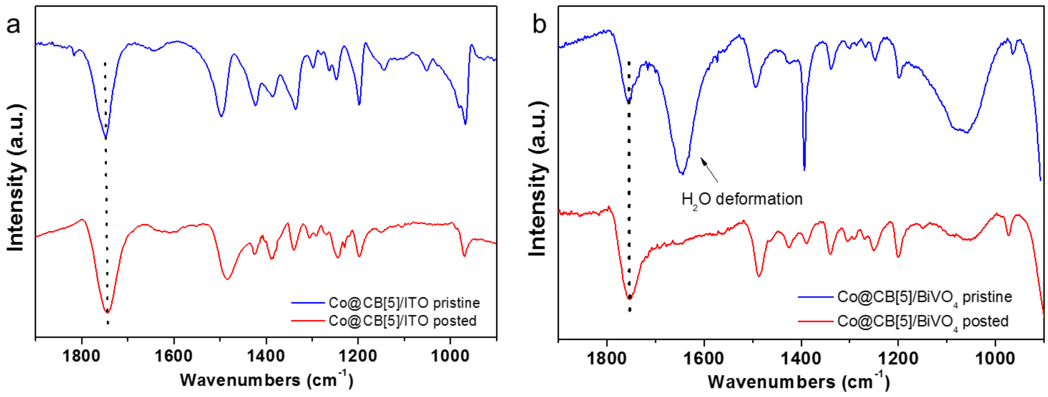

Figure S23. ATR-IR spectra of (a) Co@CB[5]/ITO and (b) Co@CB[5]/BiVO4 before and after the OER measurements.

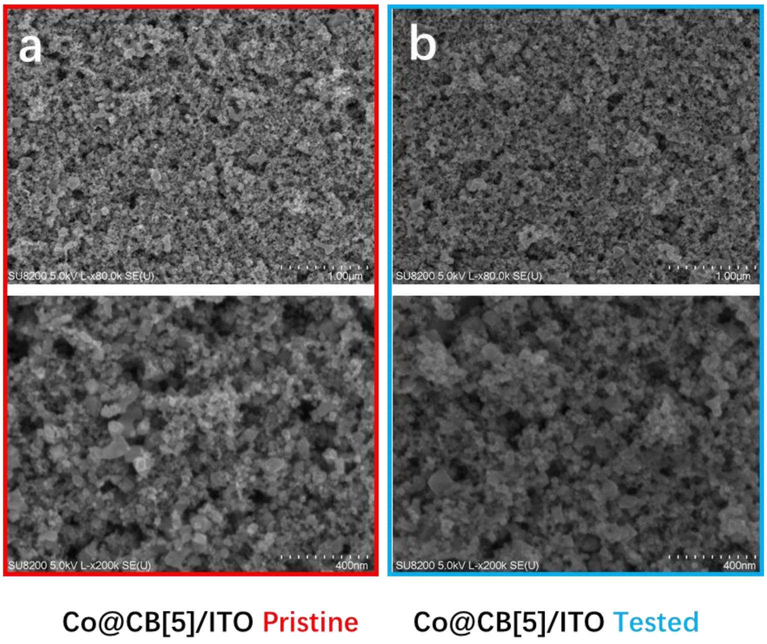

Figure S24. SEM images of Co@CB[5]/ITO. (a) pristine sample, (b) sample after OER test.

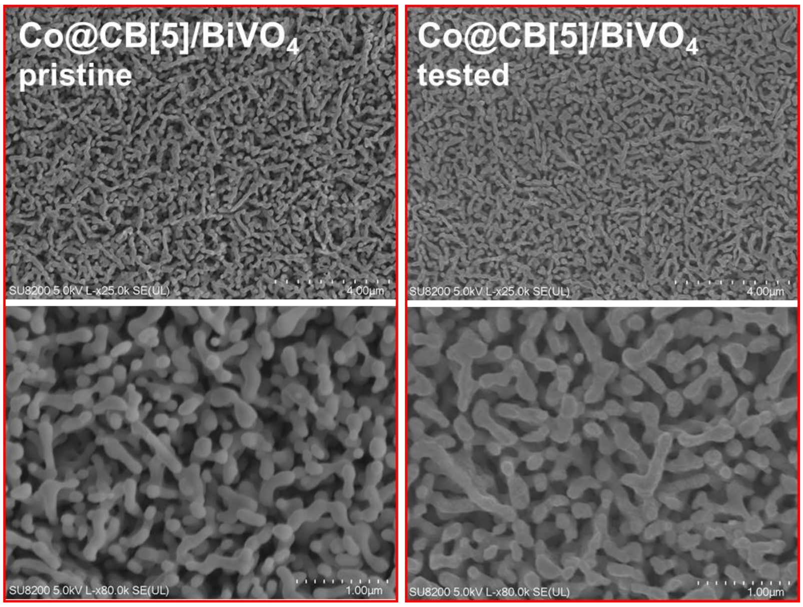

Figure S25. SEM images of Co@CB[5]/$BiVO_4$. (a) pristine sample, (b) tested sample.

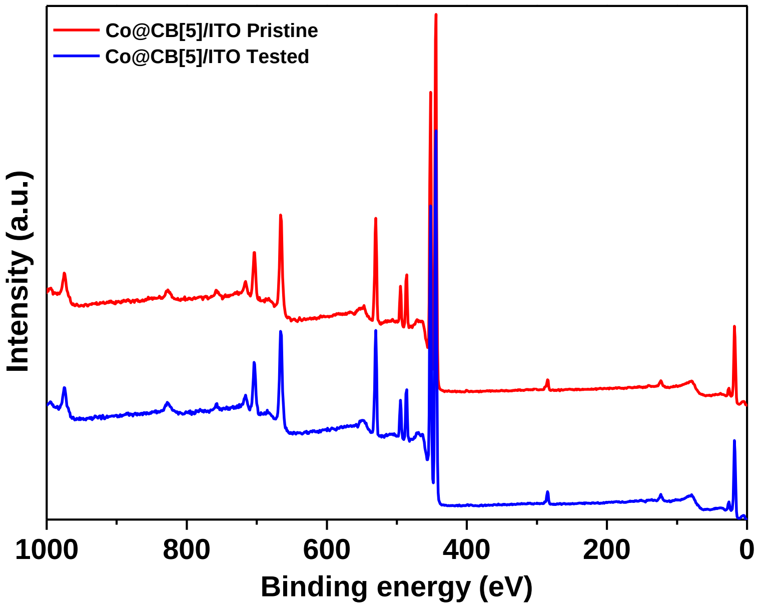

Figure S26. XPS survey spectra of pristine and tested Co@CB[5]/ITO samples.

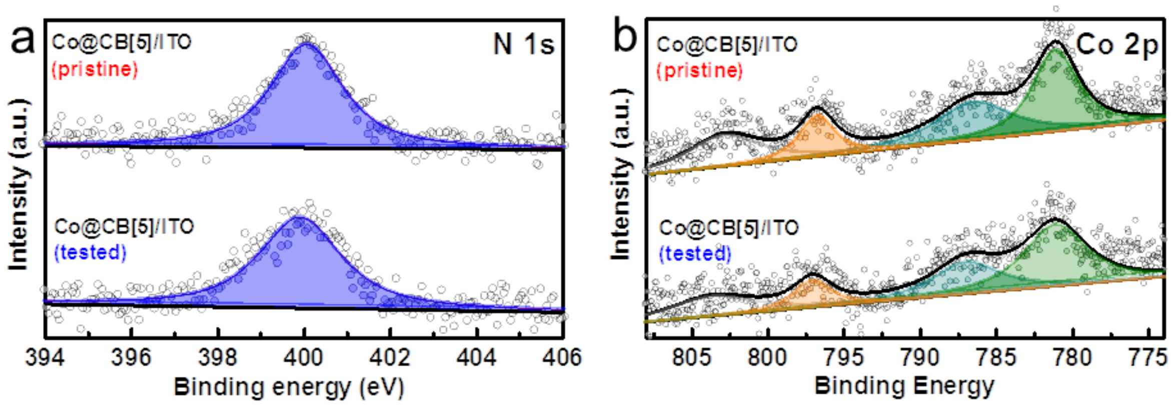

Figure S27. XPS spectra of pristine and tested Co@CB[5]/ITO in selected energy area. (a) N 1s, (b) Co 2p.

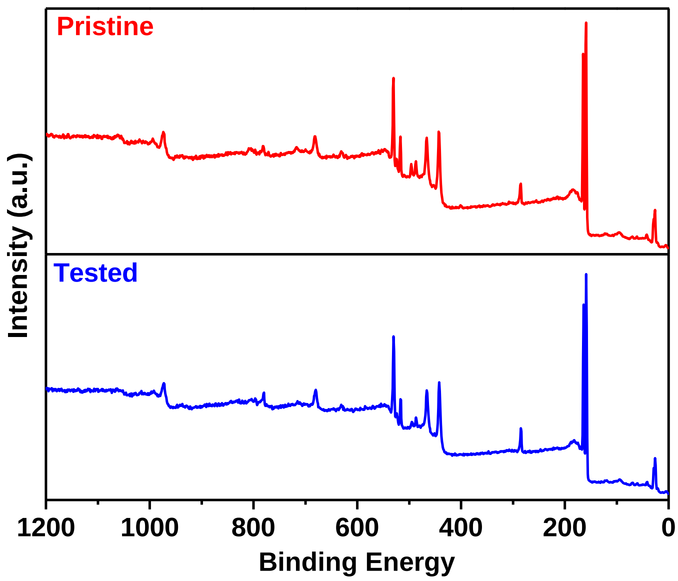

Figure S28. XPS survey spectra of pristine and tested Co@CB[5]/$BiVO_4$ samples.

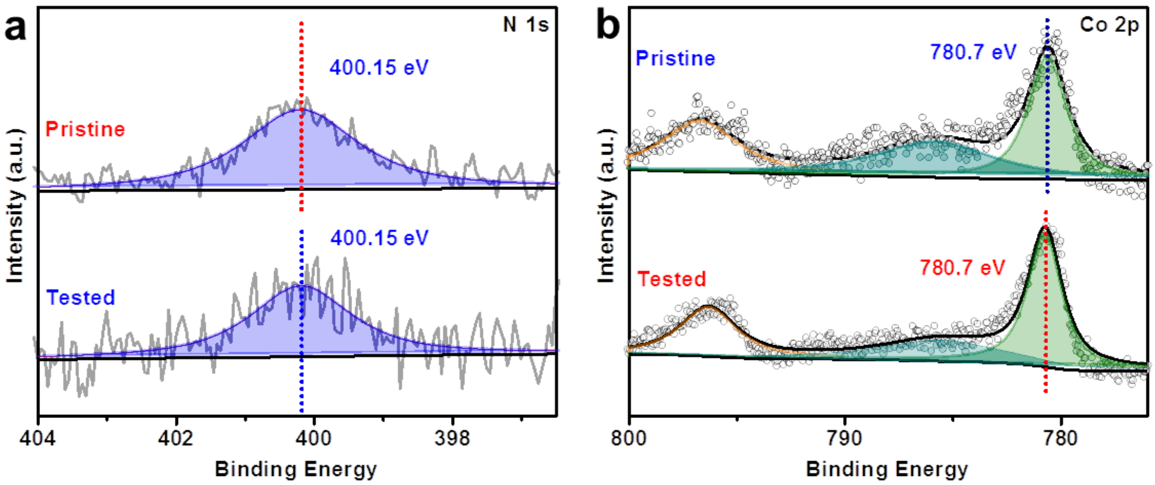

Figure S29. XPS spectra of Co@CB[5]/$BiVO_4$ photoanodes in in N 1s and Co 2p energy area before (up, Ppristine) and after (down, Tested) the OER measurements.

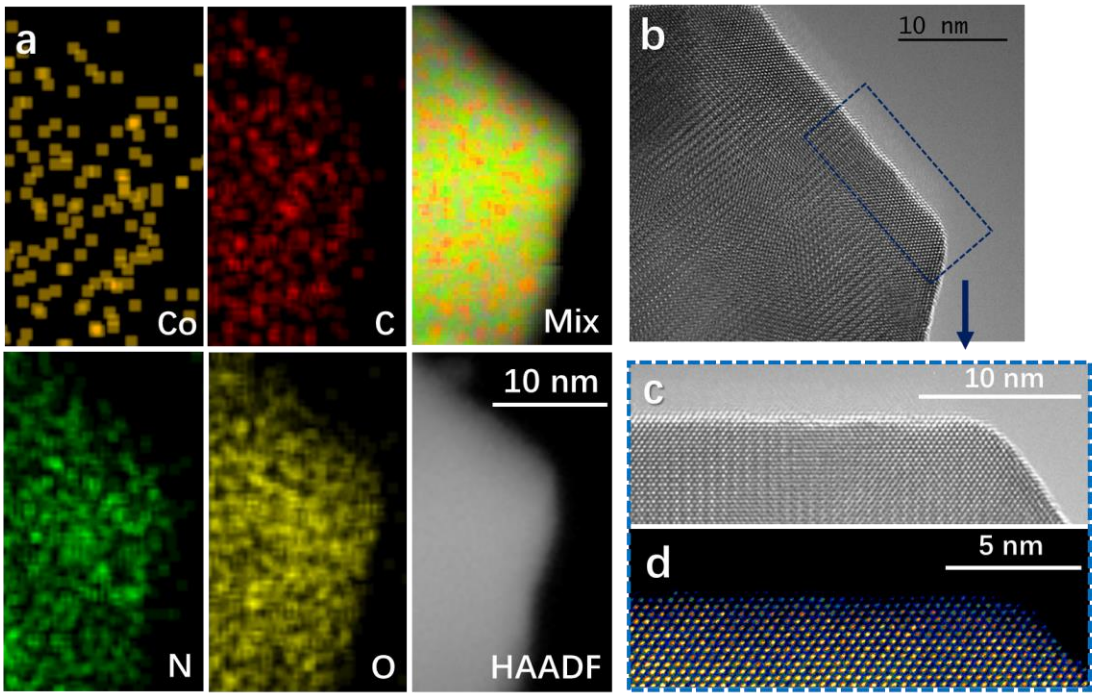

Figure S30. (a) EDS mapping images of tested Co@CB[5]/ITO particle after OER measurement. (b) Atomic-resolution TEM image of tested Co@CB[5]/ITO particle after OER measurement. TEM image (c) and corresponding HAADF image (d) in selected small area.

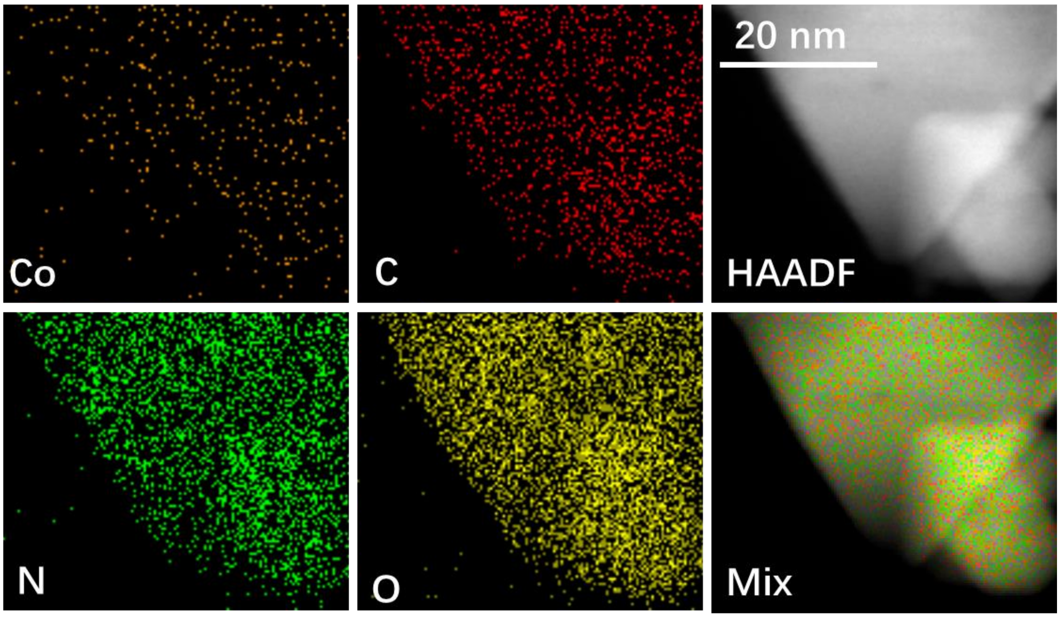

Figure S31. EDS mapping images of tested Co@CB[5]/ITO particle after OER measurement shows the homogenous distribution of Co and N over the ITO particle.

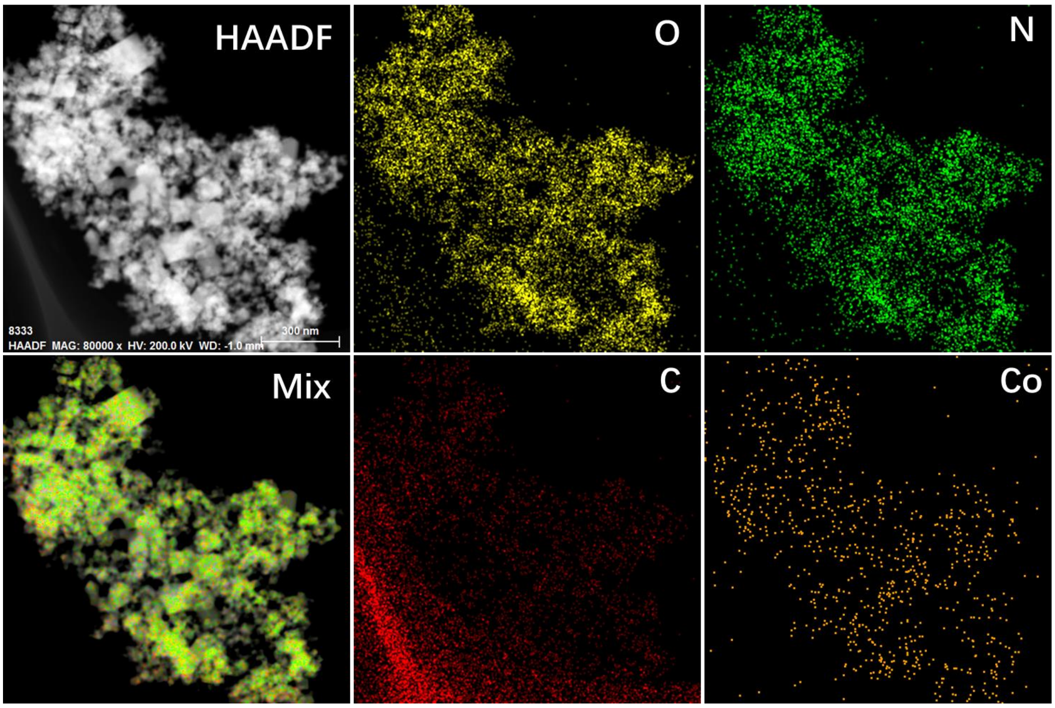

Figure S32. Large-scale EDS mapping images of tested Co@CB[5]/ITO particle after OER measurement, Co, N and C components maintain uniform distribution.

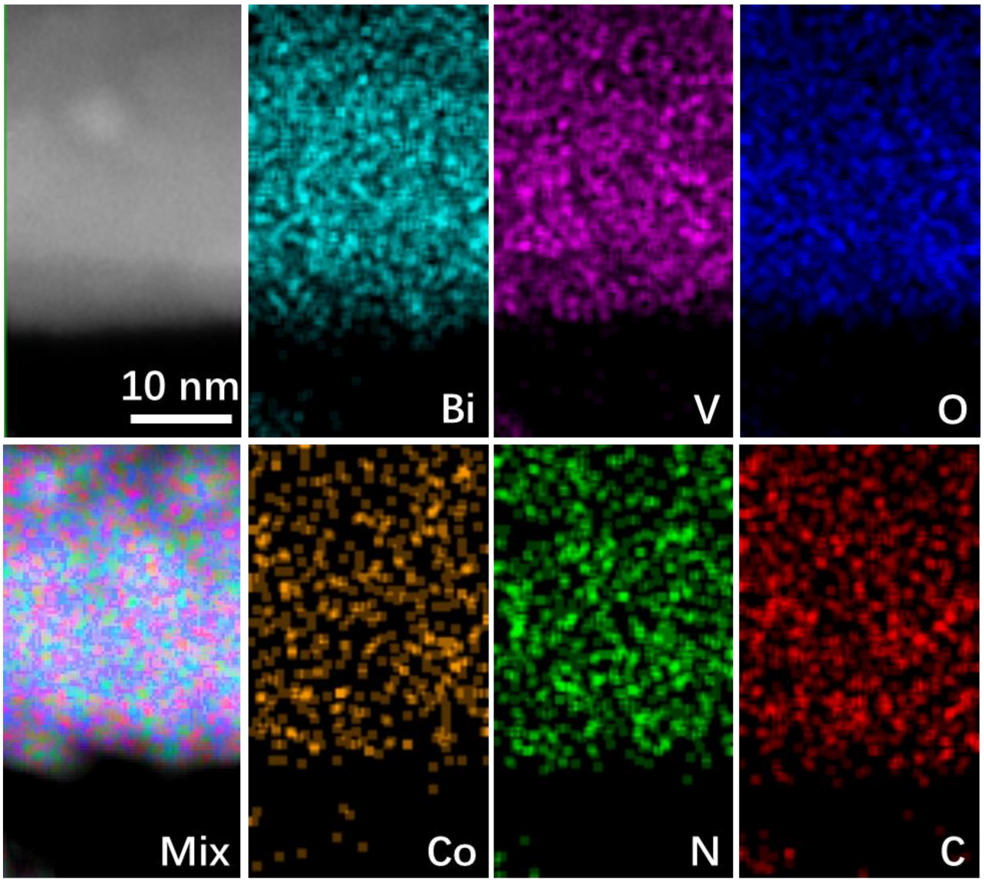

Figure S33. EDS maps of Co@CB[5]/$BiVO_4$ sample after photoelectrochemical- driven OER test. Co, N and C components maintain uniform distribution.

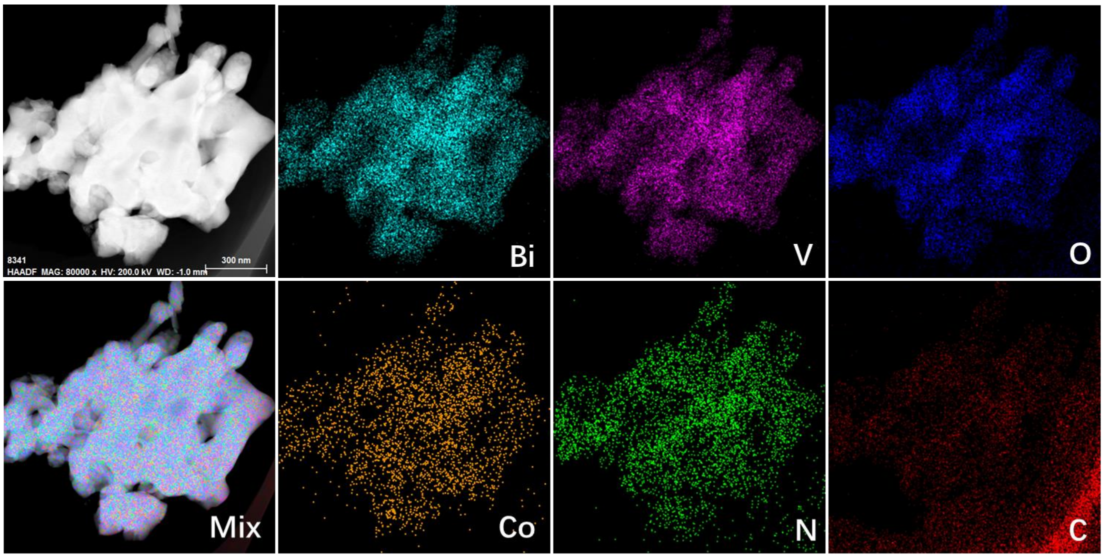

Figure S34. Large-scale EDS maps of Co@CB[5]/$BiVO_4$ sample after photoelectrochemical- driven OER test. Co, N and C components maintain uniform distribution.

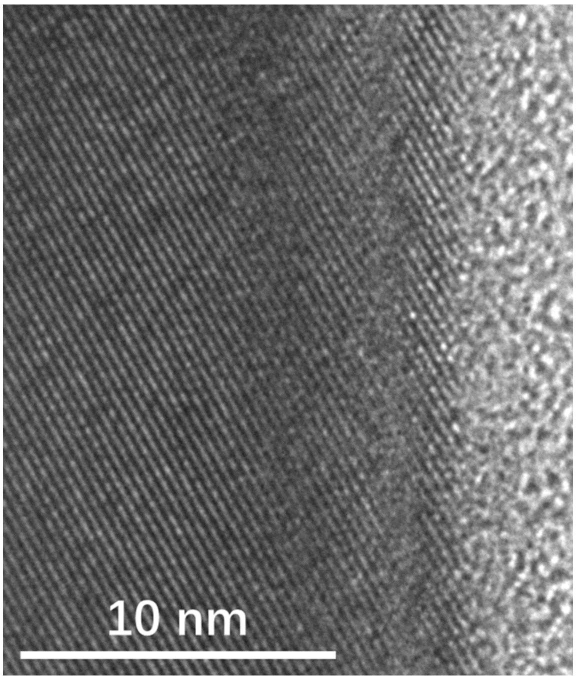

Figure S35. HRTEM image of the Co@CB[5]/$BiVO_4$ sample after photoelectrochemical driven OER test.

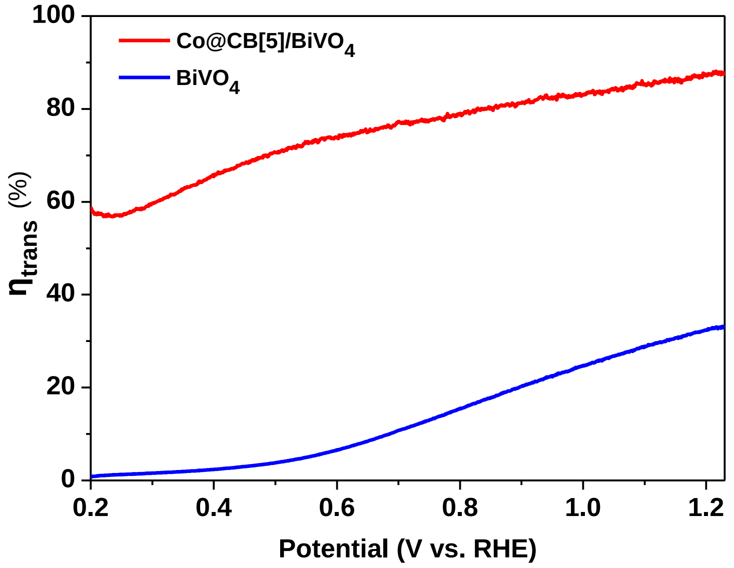

Figure S36. Surface charge transfer efficiency (η_{trans}) of the $BiVO_4$ and Co@CB[5]/$BiVO_4$ photoanodes obtained from the J–V plots of water oxidation and sulfite oxidation.

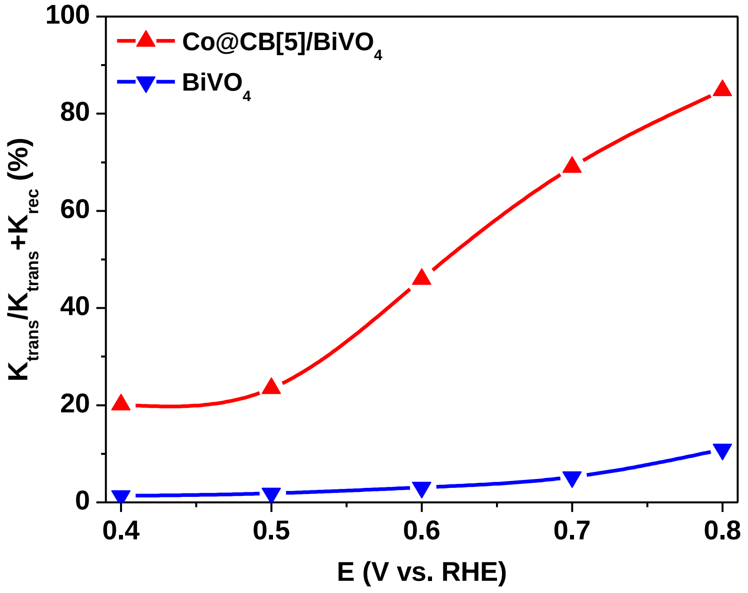

Figure S37. Surface charge transfer efficiency (η_{trans}) of the $BiVO_4$ and Co@CB[5]/$BiVO_4$ photoanodes obtained from IMPS analysis.

1. Lee, D. K.; Choi, K.-S., Enhancing long-term photostability of BiVO4 photoanodes for solar water splitting by tuning electrolyte composition. *Nat. Energy* **2017,** *3* (1), 53-60.
2. Bard, A. J.; Faulkner, L. R., *Electrochemical methods: fundamentals and applications*. Wiley New York: 1980; Vol. 2.
3. Wang, J.; Gan, L.; Zhang, W.; Peng, Y.; Yu, H.; Yan, Q.; Xia, X.; Wang, X., In situ formation of molecular Ni-Fe active sites on heteroatom-doped graphene as a heterogeneous electrocatalyst toward oxygen evolution. *Sci. Adv.* **2018,** *4* (3).
4. Bamford, C. H.; Tipper, C. F. H.; Compton, R. G., *Electrode Kinetics: Principles and Methodology: Principles and Methodology*. Elsevier: 1986.
5. Malko, D.; Kucernak, A., Kinetic isotope effect in the oxygen reduction reaction (ORR) over Fe-N/C catalysts under acidic and alkaline conditions. *Electrochem. Commun.* **2017,** *83*, 67-71.
6. Bard, A., *Standard potentials in aqueous solution*. Routledge: 2017.
7. Kohen, A.; Limbach, H.-H., *Isotope effects in chemistry and biology*. cRc Press: 2005.
